# Ruijsenaars wavefunctions as modular group matrix coefficients

**DOI:** 10.1007/s11005-024-01881-1

**Published:** 2024-11-27

**Authors:** Philippe Di Francesco, Rinat Kedem, Sergey Khoroshkin, Gus Schrader, Alexander Shapiro

**Affiliations:** 1 University of Illinois Urbana–Champaign, Champaign, IL USA; 2 Université Paris Saclay, IPHT, Gif-sur-Yvette, France; 3 National Research University Higher School of Economics, Moscow, Russia; 4 Skolkovo Institute of Science and Technology, Skolkovo, Russia; 5 Northwestern University, Evanston, IL USA; 6 University of Edinburgh, Edinburgh, UK

**Keywords:** Ruijsenaars wavefunctions, Spherical DAHA, Cluster varieties, 13F60, 17B37, 81R12

## Abstract

We give a description of the Hallnäs–Ruijsenaars eigenfunctions of the 2-particle hyperbolic Ruijsenaars system as matrix coefficients for the order 4 element $S\in SL(2,{\mathbb {Z}})$ acting on the Hilbert space of *GL*(2) quantum Teichmüller theory on the punctured torus. The *GL*(2) Macdonald polynomials are then obtained as special values of the analytic continuation of these matrix coefficients. The main tool used in the proof is the cluster structure on the moduli space of framed *GL*(2)-local systems on the punctured torus, and an $SL(2,{\mathbb {Z}})$-equivariant embedding of the *GL*(2) spherical DAHA into the quantized coordinate ring of the corresponding cluster Poisson variety.

## Introduction

In this article, we propose a description of two integrable systems, the 2-particle quantum hyperbolic Ruijsenaars system and the 2-particle *q*-difference open Toda chain, in terms of the quantum Teichmüller theory of the punctured torus. The mapping class group of the latter is isomorphic to $SL(2,\mathbb {Z})$, and understanding its action on the corresponding quantum mechanical Hilbert space enables us to reduce questions about the Ruijsenaars system into simpler ones about the Toda system. In particular, in Theorem 5.5 we obtain a description of the (suitably renormalized, *cf.* Proposition 5.2) Hallnäs–Ruijsenaars eigenfunctions $\Phi _{\varvec{\mu }}^\tau (\varvec{\lambda })$ of the hyperbolic Ruijsenaars Hamiltonians as the matrix coefficient of the element

$$\begin{aligned} S=\begin{pmatrix}0& -1\\ 1& 0\end{pmatrix}\in SL(2,{\mathbb {Z}}) \end{aligned}$$

between two states known as *q*-Whittaker functions:

1.1 $$\begin{aligned} \Phi _{\varvec{\mu }}^\tau (\varvec{\lambda }) = \left\langle \Psi _{\varvec{\lambda }}, S\Psi _{\varvec{\mu }}\right\rangle . \end{aligned}$$

The *q*-Whittaker functions $\Psi _{\varvec{\lambda }}$ are familiar objects in the well-developed algebraic and analytic theory of the open *q*-difference Toda chain, see, e.g., the following non-exhaustive list of references [8, 11, 14–17, 23, 27, 28]. In particular, as ${\varvec{\lambda }}$ ranges over ${\mathbb {R}}^2/S_2$ they furnish a complete, orthogonal set of eigendistributions for the Toda chain Hamiltonians.

Let us now discuss these integrable systems in a little more detail and outline our strategy for realizing them via quantum Teichmüller theory. The *N*-particle relativistic hyperbolic Ruijsenaars system [26] is a system of *N* commuting difference operators acting on meromorphic functions of *N* complex variables. The analytic theory of this integrable system was studied extensively in [19–21] and [1–4], where the joint eigenfunction transform was constructed and shown to define a unitary equivalence between appropriate Hilbert spaces.

The hyperbolic Ruijsenaars operators are close relatives of the Macdonald difference operators from the theory of symmetric functions. The latter operators preserve the space of $S_N$-symmetric polynomial functions, where they act diagonalizably with distinct eigenvalues, and their joint eigenfunctions are the well-known Macdonald polynomials.

As discovered by Cherednik in [6], it is useful to regard the Macdonald difference operators as generators of a commutative subalgebra in a 2-parameteric family of non-commutative algebras $\mathbb {H}_{q,t}(GL_N)$ known as the *double affine Hecke algebra* (DAHA) associated with the group $GL_N$. The DAHA is a quotient of the braid group of *N* points on a punctured 2-torus $T^2{\setminus } D^2$, which is responsible for the $SL(2,{\mathbb {Z}})$-symmetry of Macdonald theory.

In this article, we focus on the case $N=2$, in which the connection to the geometry of the torus becomes even easier to describe: the fiber at $q=1$ of the *spherical subalgebra* ${\mathbb{S}\mathbb{H}}_{q,t}$ of the $GL_2$ DAHA recovers the coordinate ring of the $GL_2({\mathbb {C}})$-character variety for the punctured torus, with the parameter *t* related to the eigenvalues of the monodromy around the puncture. In this realization of the algebra, the Macdonald operators are quantizations of the observables given by the fundamental traces of the monodromy of a *GL*(2)-local system around the (0, 1)-curve on the torus. The commuting subalgebra in ${\mathbb{S}\mathbb{H}}_{q,t}$ associated in the same way to the (1, 0)-curve is identified with that generated by the operators of multiplication by the elementary symmetric functions acting on the ring of 2-variable symmetric polynomials.

To reconnect this algebraic picture with the analytic Ruijsenaars theory, we recall from the work of Fock and Goncharov [13] that the punctured torus character variety (or more precisely, a decorated version thereof, where the local systems are equipped with extra data of a Borel reduction at the puncture) is a cluster Poisson variety. Hence, its quantization delivers a (projective) unitary Hilbert space representation of the torus mapping class group $SL(2,\mathbb {Z})$, which acts by explicit quantum cluster transformations. The (0, 1)-curve generators of the spherical DAHA ${\mathbb{S}\mathbb{H}}_{q,t}$, which correspond to the Macdonald–Ruijsenaars operators in Cherednik’s representation, act by unbounded, symmetric operators on this Hilbert space. On the other hand, we show the (1, 0)-curve generators act in the cluster representation by the quantum open Toda chain Hamiltonians, which leads directly to formula (1.1). The matrix coefficient realization of the Hallnäs–Ruijsenaars eigenfunctions makes immediate most of their important properties, including the “bispectral” and “Poincaré duality” symmetries $\Phi _{\varvec{\lambda }}^{\tau }({\varvec{\mu }})=\Phi _{\varvec{\mu }}^\tau ({\varvec{\lambda }})$ and $\Phi _{\varvec{\mu }}^{-\tau }({\varvec{\lambda }})=\Phi _{\varvec{\lambda }}^\tau ({\varvec{\mu }})$, see Corollaries 5.3 and 5.6.

From the physical point of view, the formula (1.1) expresses the Hallnäs–Ruijsenaars eigenfunctions as scalar products between eigenstates of two quantum mechanical integrable systems given by traces of holonomies along the (1, 0) and (0, 1)-curves on the torus, respectively. In other words, the Hallnäs–Ruijsenaars eigenfunction is the transition matrix between two geometrically natural bases in the Hilbert space for quantum Teichmüller theory on $T^2{\setminus } D^2$. This transition matrix realizes the action of the *S*-move generator in the corresponding projective representation of the Moore–Seiberg groupoid and has been previously computed by Teschner and Vartanov, see formula (6.30) of [33].

In this sense, formula (1.1) is quite analogous to the well-known description of the $SU_2$ Racah–Wigner *q*-6*j* symbols which arises in an almost identical way, except that the punctured torus is replaced by the 4-punctured sphere. The semiclassical asymptotics of such scalar products were described geometrically in [25], where the two classical integrable systems determine two presentations of the character variety as Lagrangian fibrations, and the asymptotic expansion of the scalar product takes the form of a sum over intersection points of the two corresponding Lagrangian fibers.

In Sect. 6, we obtain *q*-Whittaker and Macdonald polynomials, respectively, as special values of analytically continued *q*-Whittaker and Hallnäs–Ruijsenaars eigenfunctions. In a similar fashion, we also recover the Harish–Chandra series solutions for the 2-particle quantum open Toda chain and the 2-particle quantum hyperbolic Ruijsenaars system. We conclude this article with a discussion of the *N*-particle case, which we shall return to in a separate publication.

## Ruijsenaars and open Toda integrable systems

In this section, we recall the definition of the two integrable systems that play a central role in the paper: the two-particle hyperbolic Ruijsenaars system and the open *q*-difference Toda chain.

### Ruijsenaars system

The ${\mathfrak {gl}}_2$ Macdonald difference operators

$$\begin{aligned} M_j({\varvec{\lambda }}; g \,|\,{\varvec{\omega }}),\qquad j=1,2 \end{aligned}$$

with periods $\omega _1, \omega _2$ and the coupling constant *g* are the commuting operators^1^

$$\begin{aligned} M_1({\varvec{\lambda }}; g \,|\,{\varvec{\omega }})&= \frac{\operatorname {sh}\frac{\pi }{\omega _2}\left( \lambda _1-\lambda _2+i g\right) }{\operatorname {sh}\frac{\pi }{\omega _2}\left( \lambda _1-\lambda _2\right) } e^{i\omega _1\frac{\partial }{\partial \lambda _1}} + \frac{\operatorname {sh}\frac{\pi }{\omega _2}\left( \lambda _2-\lambda _1+i g\right) }{\operatorname {sh}\frac{\pi }{\omega _2}\left( \lambda _2-\lambda _1\right) } e^{i\omega _1\frac{\partial }{\partial \lambda _2}}, \\ M_2({\varvec{\lambda }}; g \,|\,{\varvec{\omega }})&= e^{i\omega _1\left( \frac{\partial }{\partial \lambda _1}+\frac{\partial }{\partial \lambda _2}\right) }, \end{aligned}$$

where we write ${\varvec{x}}$ for a vector $(x_1,x_2) \in \mathbb {R}^2$. Given $b\in {\mathbb {R}}_{>0}$, let us specialize

2.1 $$\begin{aligned} \omega _1 = b, \qquad \omega _2=b^{-1} \end{aligned}$$

and introduce the pure imaginary constant

$$\begin{aligned} c_b = \frac{i(b + b^{-1})}{2}. \end{aligned}$$

We will work in the regime in which the coupling constant *g* admits a parametrization

2.2 $$\begin{aligned} g = -i(c_b+2\tau ) \qquad {\text {for}}\qquad \tau \in \mathbb {R}. \end{aligned}$$

Then, for $j=1,2$ we recover the Macdonald operators $M^{\tau }_j = M_j({\varvec{\lambda }}; -i(c_b+2\tau ) \,|\,b, b^{-1})$, which take the form

$$\begin{aligned} M^{\tau }_1&= \frac{t\Lambda _1 - t^{-1}\Lambda _2}{\Lambda _1-\Lambda _2}T_{\Lambda _1} + \frac{t\Lambda _2 - t^{-1}\Lambda _1}{\Lambda _2-\Lambda _1}T_{\Lambda _2}, \\ M^{\tau }_2&= T_{\Lambda _1}T_{\Lambda _2}, \end{aligned}$$

with

$$\begin{aligned} \Lambda _j = e^{2\pi b\lambda _j},\qquad T_{\Lambda _j} = e^{ib\frac{\partial }{\partial \lambda _j}}, \qquad {\text {and}}\qquad t = e^{\pi b (2\tau +c_b)}. \end{aligned}$$

The operators $T_{\Lambda _j}$ act on functions $f({\varvec{\Lambda }})$ as shift operators:

$$\begin{aligned} T_{\Lambda _1} f(\Lambda _1,\Lambda _2) = f(q^2\Lambda _1,\Lambda _2), \qquad T_{\Lambda _2} f(\Lambda _1,\Lambda _2) = f(\Lambda _1,q^2\Lambda _2) \end{aligned}$$

where

$$\begin{aligned} q = e^{\pi i b^2}. \end{aligned}$$

The Macdonald difference operators $M^{\tau }_j$ are essentially self-adjoint with respect to the Hermitian inner product $\left\langle \cdot ,\cdot \right\rangle _S$ on $L^2_{\textrm{sym}}(\mathbb {R}^2, m({\varvec{\lambda }})d{\varvec{\lambda }})$ defined by

2.3 $$\begin{aligned} \left\langle f,g\right\rangle _{Sk}=\int _{\mathbb {R}^2} \overline{f({\varvec{\lambda }})} g({\varvec{\lambda }})m({\varvec{\lambda }})d{\varvec{\lambda }}, \end{aligned}$$

where

2.4 $$\begin{aligned} m({\varvec{\lambda }})d{\varvec{\lambda }} = 2\operatorname {sh}(b(\lambda _1-\lambda _2))\operatorname {sh}(b^{-1}(\lambda _1-\lambda _2))d{\varvec{\lambda }} \end{aligned}$$

is the ${\mathfrak {gl}}_2$ Sklyanin measure.

Joint eigenfunctions of Macdonald operators were obtained in [19] and further studied in [20, 21], as well as [1–4]. In the specialization (2.1), (2.2), these eigenfunctions take form

2.5 $$\begin{aligned} \textrm{P}^\tau _{\varvec{\mu }}({\varvec{\lambda }}) = \varphi (c_b-2\tau _-) e^{-\pi i \underline{\varvec{\lambda }} \underline{\varvec{\mu }}} \int _\mathbb {R}e^{-4\pi i x(\lambda +\tau _-)} \frac{\varphi (x+\mu +\tau _-) \varphi (x-\mu +\tau _-)}{\varphi (x+\mu -\tau _-) \varphi (x-\mu -\tau _-)} dx, \end{aligned}$$

where $\varphi (z)$ is the *non-compact quantum dilogarithm* discussed in Appendix,

$$\begin{aligned} \tau _- = \frac{c_b}{2}-\tau , \qquad a = \frac{a_1-a_2}{2}, \qquad \underline{{\varvec{a}}} = a_1+a_2 \qquad {\text {for}}\qquad a = \lambda ,\mu . \end{aligned}$$

As shown in [2], $\textrm{P}^\tau _{\varvec{\mu }}({\varvec{\lambda }})$ are symmetric in the coordinate variables ${\varvec{\lambda }}$, symmetric in the spectral variables ${\varvec{\mu }}$, and satisfy the bispectral duality

$$\begin{aligned} \textrm{P}^\tau _{\varvec{\mu }}({\varvec{\lambda }}) = \textrm{P}^{-\tau }_{\varvec{\lambda }}({\varvec{\mu }}). \end{aligned}$$

Using the asymptotics (A.5) of the function $\varphi (z)$ as $\operatorname {Re}(z)\rightarrow \pm \infty$ described in Appendix, one sees that the integral (2.5) converges whenever

$$\begin{aligned} \operatorname {Im}(\tau \pm \lambda )<\frac{b+b^{-1}}{4}. \end{aligned}$$

The Macdonald difference operators also act on the space of $\mathbb {C}(q,t)$-valued Laurent polynomials in $\Lambda$, invariant under the involution $\Lambda \mapsto \Lambda ^{-1}$. Their eigenbasis in the latter space is given by the celebrated symmetric Macdonald polynomials, which can be recovered as the Hallnäs–Ruijsenaars wavefunctions restricted to a lattice, see Section 6 for more details.

#### Remark 2.1

Consider the Macdonald–Ruijsenaars measure

$$\begin{aligned} m_g({\varvec{\lambda }}) = S_2(i(\lambda _1-\lambda _2)) S_2(i(\lambda _2-\lambda _1)+g), \end{aligned}$$

where $S_2$ is the Barnes double sine function from equation (A.9). The corresponding bilinear pairing $\left( \cdot ,\cdot \right) _R$ and Hermitian pairing $\left\langle \cdot ,\cdot \right\rangle _R$ are defined by

$$\begin{aligned} \left( f,g\right) _R = \int _{\mathbb {R}^2}f({\varvec{\lambda }})g(-{\varvec{\lambda }})m_g({\varvec{\lambda }}) d{\varvec{\lambda }}, \qquad \left\langle f,g\right\rangle _R = \int _{\mathbb {R}^2}f({\varvec{\lambda }})\overline{g({\varvec{\lambda }})}m_g({\varvec{\lambda }}) d{\varvec{\lambda }}. \end{aligned}$$

**Fig. 1:**
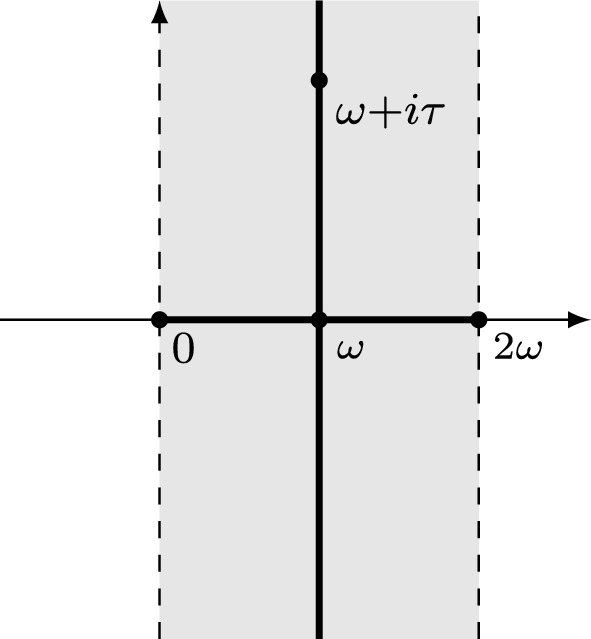
Regions of unitarity of the Ruijsenaars system in the *g*-plane

As shown in, e.g., [2], for any periods $\omega _1, \omega _2$, and the coupling constant *g* in the strip of analyticity of the Ruijsenaars system, that is, $0< \operatorname {Re}(g) < \omega _1+\omega _2$, the Macdonald operators are essentially self-adjoint with respect to the bilinear pairing $\left( \cdot ,\cdot \right) _R$. Moreover, as shown in [3], when the coupling constant *g* is real, the Hallnäs–Ruijsenaars functions are orthogonal and complete with respect to the pairing $\left\langle \cdot ,\cdot \right\rangle _R$. It is immediate from (A.1) and (A.6) that for $\tau \in i\mathbb {R}$ the Hallnäs–Ruijsenaars functions satisfy

$$\begin{aligned} \textrm{P}_{\varvec{\mu }}^\tau (-{\varvec{\lambda }}) = \zeta _{inv} e^{4\pi i \tau ^2} \overline{\textrm{P}_{\varvec{\mu }}^\tau ({\varvec{\lambda }})}, \end{aligned}$$

where the constant $\zeta _{inv}$ is given by (A.2), and hence,

$$\begin{aligned} \left( \textrm{P}_{\varvec{\mu }}^\tau ,\textrm{P}_{\varvec{\nu }}^\tau \right) _R = \zeta _{inv} e^{4\pi i \tau ^2} \left\langle \textrm{P}_{\varvec{\mu }}^\tau ,\textrm{P}_{\varvec{\nu }}^\tau \right\rangle _R. \end{aligned}$$

Thus, we have two regimes of unitarity of the Ruijsenaars system, and Macdonald operators are symmetric in both of them. In the first regime, the coupling constant *g* is real and satisfies $0< g < -ic_b$, equivalently $\tau$ is imaginary with $\left| \tau \right| < c_b/2$, and the scalar product is $\left\langle \cdot ,\cdot \right\rangle _R$. In the second regime $g = i(\tau -c_b)$ with an arbitrary real $\tau$, and the scalar product is $\left\langle \cdot ,\cdot \right\rangle _{Sk}$, see (2.3). In [19–21] and [1–4], the Ruijsenaars system was studied in the first regime. In the present text, we focus on the second. In Fig. 1, we visualize the two regimes for $\omega _1=b$, $\omega _2=b^{-1}$, and $\omega =-ic_b$.

### Open Toda chain

The Hamiltonians of the *q*-difference ${\mathfrak {gl}}_2$ open Toda system are the following commuting difference operators

2.6 $$\begin{aligned} \begin{aligned} H_1&= e^{2\pi b p_2} + e^{2\pi b(p_2+x_2-x_1)} + e^{2\pi b p_1}, \\ H_2&= e^{2\pi b(p_1+p_2)}, \end{aligned} \end{aligned}$$

where

$$\begin{aligned} p_j = \frac{1}{2\pi i}\frac{\partial }{\partial x_j}. \end{aligned}$$

These operators are evidently symmetric with respect to the Hermitian inner product $\left\langle \cdot ,\cdot \right\rangle$ on $L^2(\mathbb {R}^2, d{\varvec{x}})$ given by

2.7 $$\begin{aligned} \left\langle f,g\right\rangle =\int _{\mathbb {R}^2} \overline{f({\varvec{x}})} g({\varvec{x}}) d{\varvec{x}}. \end{aligned}$$

They also commute with the *Q*-*system discrete time evolution operator* [9, 10, 28] (see also [18, 34] for the discussion of classical *Q*-system), which is the unitary automorphism *D* of $L^2(\mathbb {R}^2, d{\varvec{x}})$ given by

2.8 $$\begin{aligned} D = e^{\pi i\left( p_1^2+p_2^2\right) } \varphi (x_2-x_1). \end{aligned}$$

#### Remark 2.2

In Sect. 4, the *Q*-system evolution operator *D* will be re-interpreted as a Dehn twist $\sigma _+$ on the punctured torus. See Remark 4.1 for details.

For ${\varvec{\lambda }} \in {\mathbb {R}}^2$, the *Whittaker function* $\Psi _{\varvec{\lambda }}({\varvec{x}})$ is a common eigenfunction for the operators $H_1$, $H_2$, and *D* with the following eigenvalues, see [23, 28]:

2.9 $$\begin{aligned} \begin{aligned} H_j \Psi _{\varvec{\lambda }}({\varvec{x}})&= e_j({\varvec{\lambda }}) \Psi _{\varvec{\lambda }}({\varvec{x}}), \\ D \Psi _{\varvec{\lambda }}({\varvec{x}})&= \gamma \Psi _{\varvec{\lambda }}({\varvec{x}}), \end{aligned} \end{aligned}$$

where $e_j(\lambda )$ is the *j*-th elementary symmetric function in $e^{2\pi b \lambda _1}$, $e^{2\pi b \lambda _2}$, and

2.10 $$\begin{aligned} \gamma = e^{\pi i\left( \lambda _1^2+\lambda _2^2\right) }. \end{aligned}$$

The Whittaker function admits two explicit presentations, both of which utilize the following convention.

#### Notation 2.3

Throughout the paper, we will often consider contour integrals of the form

$$\begin{aligned} \int _{C} \prod _{j,k}\frac{\varphi (t-a_j)}{\varphi (t-b_k)}f(t)dt, \end{aligned}$$

where *f*(*t*) is some entire function. Unless otherwise specified, the contour *C* in such an integral is always chosen to be passing below the poles of $\varphi (t-a_j)$ for all *j*, above the poles of $\varphi (t-b_k)^{-1}$ for all *k*, and escaping to infinity in such a way that the integrand is rapidly decaying.

The Gauss–Givental representation of the Whittaker function reads

2.11 $$\begin{aligned} \Psi _{\varvec{\lambda }}({\varvec{x}}) = \zeta ^{-1} e^{\pi i c_b (\lambda _2-\lambda _1)} e^{2\pi i \lambda _2 \underline{{\varvec{x}}}} \int \frac{e^{2\pi i r(\lambda _1-\lambda _2) }}{\varphi (r-x_1-c_b)\varphi (x_2-r)}dr, \end{aligned}$$

where $\zeta$ is defined in (A.2), and

$$\begin{aligned} \underline{{\varvec{x}}} = x_1+x_2. \end{aligned}$$

On the other hand, the function $\Psi _{\varvec{\lambda }}({\varvec{x}})$ can also be expressed as a Mellin–Barnes integral:

2.12 $$\begin{aligned} \Psi _{\varvec{\lambda }}({\varvec{x}}) = \zeta e^{\pi i \underline{\varvec{\lambda }}(2x_2+c_b)} \int e^{2\pi i \alpha (x_1-x_2-c_b)} \varphi (\alpha -\lambda _1+c_b) \varphi (\alpha -\lambda _2+c_b) d\alpha . \end{aligned}$$

It is manifest from the Mellin–Barnes presentation that $\Psi _{\varvec{\lambda }}({\varvec{x}})$ is symmetric in the spectral variables ${\varvec{\lambda }}$. The following result is fundamental for the rest of the paper. Its higher rank generalization was established in [28].

#### Theorem 2.4

([22]) Let $\Psi  _{\varvec{\lambda }}({\varvec{x}})$ be the Whittaker function for the ${\mathfrak {gl}}_2$ *q*-difference open Toda chain. Define the *Whittaker transform* by

$$\begin{aligned} \mathcal {W}:L^2(\mathbb {R}^2, d{\varvec{x}})&\longrightarrow L^2_{\textrm{sym}}(\mathbb {R}^2, m({\varvec{\lambda }})d{\varvec{\lambda }}) \\ f({\varvec{x}})&\longmapsto \int _{\mathbb {R}^n} f({\varvec{x}}) \overline{\Psi _{\varvec{\lambda }} ({\varvec{x}})} d{\varvec{x}}. \end{aligned}$$

Then, ${\mathcal {W}}$ defines a unitary equivalence between the Hilbert space of square-integrable functions in ${\varvec{x}}$ and that of symmetric functions in ${\varvec{\lambda }}$ that are square-integrable with respect to the Sklyanin measure $m({\varvec{\lambda }})$ given by (2.4).

The Whittaker functions $\Psi _{\varvec{\lambda }}({\varvec{x}})$ also satisfy difference equations with respect to the spectral variables ${\varvec{\lambda }}$. Indeed, in terms of the *dual Toda operators* $\check{H}_j$ defined by

$$\begin{aligned} {\check{H}}_1&= \left( tM^{\tau }_1\right) \Big |_{t=0} = \frac{1}{1-\Lambda _1/\Lambda _2}T_{\Lambda _1} + \frac{1}{1-\Lambda _2/\Lambda _1}T_{\Lambda _2}, \\ {\check{H}}_2&= M^{\tau }_2, \end{aligned}$$

the bi-spectrality of the Whittaker functions can be expressed as follows: (see [29, Theorem 4.7])

$$\begin{aligned} \mathcal {W}\circ H_1&= e_1({\varvec{\lambda }}) \circ \mathcal {W},&\mathcal {W}\circ e^{\pi b c_b} e^{2\pi b x_1}&= {\check{H}}_1 \circ \mathcal {W}, \\ \mathcal {W}\circ H_2&= e_2({\varvec{\lambda }}) \circ \mathcal {W},&\mathcal {W}\circ e^{2\pi b(x_1+x_2)}&= {\check{H}}_2 \circ \mathcal {W}. \end{aligned}$$

It is also immediate from (2.9) that the *Q*-system evolution is intertwined under Whittaker transform with the multiplication operator $\gamma$:

2.13 $$\begin{aligned} {\mathcal {W}}\circ D = \gamma \circ {\mathcal {W}}. \end{aligned}$$

Following [9], let us define the *n*-th discrete time translate ${\check{H}}_{j,n}$ of ${\check{H}}_{j,0}={\check{H}}_j$ by

2.14 $$\begin{aligned} {\check{H}}_{j,n} = \gamma ^n {\check{H}}_j \gamma ^{-n}. \end{aligned}$$

Using that $\gamma T_{\Lambda _j} \gamma ^{-1} = q \Lambda _j T_{\Lambda _j}$, we obtain

$$\begin{aligned} {\check{H}}_{1,n} = q^n\left( \frac{\Lambda _1^n}{1-\Lambda _1/\Lambda _2}T_{\Lambda _1} + \frac{\Lambda _2^n}{1-\Lambda _2/\Lambda _1}T_{\Lambda _2}\right) \end{aligned}$$

and recalling the intertwining relation (2.13) we have

$$\begin{aligned} {\check{H}}_{1,n} \circ \mathcal {W}= \mathcal {W}\circ \left( e^{\pi b c_b} \cdot D^n e^{2\pi b x_1} D^{-n}\right) . \end{aligned}$$

Note that by collecting coefficients in *t*, the first Macdonald operator $M^{\tau }_1$ can be expanded as a linear combination of translates of dual Toda operators:

$$\begin{aligned} M^{\tau }_1 = t^{-1}{\check{H}}_{1,0} - q^{-2}\frac{t}{\Lambda _1\Lambda _2} \check{H}_{1,2}. \end{aligned}$$

As a consequence, we obtain the following intertwining relations:

2.15 $$\begin{aligned} M_1^\tau \circ \mathcal {W}&= \mathcal {W}\circ \left( e^{2\pi b(x_1-\tau )} + e^{2\pi b(x_2+\tau )} + e^{2\pi b(x_1+p_1-p_2+\tau )}\right) , \nonumber \\ M_2^\tau \circ \mathcal {W}&= \mathcal {W}\circ e^{2\pi b(x_1+x_2)}. \end{aligned}$$

These relations can be interpreted as describing the action of the Macdonald operators $M_j^\tau$ in the basis of Whittaker functions $\Psi _{\varvec{\lambda }}({\varvec{x}})$, where the latter are regarded as functions of ${\varvec{\lambda }}$.

### Spherical double affine Hecke algebra

Recall that the elliptic braid group is the fundamental group of the configuration space of *N* points on the torus $T^2$. The double affine Hecke algebra $\mathbb {H}_{q,t}$ for *GL*(*N*) is the quotient of the ${\mathbb {Q}}(q,t)$-group algebra of the elliptic braid group by the Hecke relations

$$\begin{aligned} (T_i - t)(T_i + t^{-1}) = 0, \quad i=1,\ldots N, \end{aligned}$$

where $T_i$ are the standard “local” generators corresponding to those of the planar braid group. In addition to these local generators, there are also “global” ones, $X_i$ and $Y_i$, corresponding to the loops in the configuration space transporting the *i*-th marked point once along, respectively, the (1, 0)- and the (0, 1)-curves on the torus. Being a quotient of the elliptic braid group, the algebra $\mathbb {H}_{q,t}$ carries an action of the modular group $SL(2;{\mathbb {Z}})$ by algebra automorphisms.

We skip the precise definition of the *spherical subalgebra* ${\mathbb{S}\mathbb{H}}_{q,t}$ of $\mathbb {H}_{q,t}$ and refer the reader to [6] for details. Instead, following *loc. cit.* we recall that ${\mathbb{S}\mathbb{H}}{q,t}$ admits a faithful representation into the algebra of symmetric *q*-difference operators in variables $(\Lambda _1,\Lambda _2)$. The spherical subalgebra contains elements $E_{(\pm k,0)}, E_{(0,\pm k)}$ corresponding to spherical versions of the *k*-th power sum symmetric functions in the standard generators $X^{\pm }_i,Y^{\pm }_i$ of $\mathbb {H}_{q,t}$, respectively. As shown in [31], the algebra ${\mathbb{S}\mathbb{H}}_{q,t}$ is generated over ${\mathbb {Q}}(q,t)$ by elements $E_{(\pm 1,0)}, E_{(0,\pm 1)}$, see [5, Corollary 6.1].

Under Cherednik’s representation, these generators are mapped to the following *q*-difference operators:

$$\begin{aligned} E_{(1,0)}&\longmapsto e_1({\varvec{\lambda }}),&E_{(-1,0)}&\longmapsto e_1({\varvec{\lambda }})e_2({\varvec{\lambda }})^{-1}, \\ E_{(0,1)}&\longmapsto M^{\tau }_1,&E_{(0,-1)}&\longmapsto M^{\tau }_1\left( M^{\tau }_2\right) ^{-1}. \end{aligned}$$

The action of $SL(2;{\mathbb {Z}})$ preserves the spherical subalgebra: For each $v\in {\mathbb {Z}}^2$, there is a corresponding element $E_v\in {\mathbb{S}\mathbb{H}}_{q,t}$, and we have $g\cdot E_v = E_{gv}$ for all $g\in SL(2;{\mathbb {Z}}),~v\in {\mathbb {Z}}^2$.

### Strategy

Let us now outline in more detail the logic of the remainder of the paper. The double affine Hecke algebra formalism suggests that the problem of diagonalizing the Macdonald difference operators can be solved by constructing a unitary representation $\rho$ of $SL(2;{\mathbb {Z}})$ on $L^2_{\textrm{sym}}(\mathbb {R}^n, m({\varvec{\lambda }})d{\varvec{\lambda }})$ compatible with its action on the spherical DAHA. Indeed, the action of the element *S* would then intertwine the Macdonald operators with the operators of multiplication by elementary symmetric functions:

$$\begin{aligned} M^{\tau }_j \circ \rho (S) = \rho (S) \circ e_j({\varvec{\lambda }}). \end{aligned}$$

The eigenbasis for the latter is given by delta-distributions. Here, we must note that while delta-distributions are clearly not in $L^2_{\textrm{sym}}(\mathbb {R}^n, m({\varvec{\lambda }})d{\varvec{\lambda }})$, it is only expected that the eigenfunctions of an unbounded operator lie in the space of tempered distributions rather than in the Hilbert space itself. Thus, the eigenbasis for the Macdonald operator would consist of the images of delta-distributions under the *S* transformation:

$$\begin{aligned} M^{\tau }_j \rho (S) \delta ({\varvec{\lambda }}-{\varvec{\mu }}) = e_j({\varvec{\mu }}) \cdot \rho (S) \delta ({\varvec{\lambda }}-{\varvec{\mu }}). \end{aligned}$$

Our strategy for constructing the action of $SL(2;{\mathbb {Z}})$ on $L^2_{\textrm{sym}}(\mathbb {R}^n, m({\varvec{\lambda }})d{\varvec{\lambda }})$ is to identify the latter with the unitary equivalent representation $L^2(\mathbb {R}^n, d\varvec{x})$ via the Whittaker transform $\mathcal {W}$. As we will show, the action of generators of ${\mathbb{S}\mathbb{H}}_{q,t}$ on $L^2(\mathbb {R}^n, d{\varvec{x}})$ coincides with that of certain elements of the quantized coordinate ring of a *cluster Poisson variety*. The representation of the latter comes equipped with a compatible action of the group $SL(2;{\mathbb {Z}})$, which acts on $L^2(\mathbb {R}^n, d{\varvec{x}})$ by explicit unitary quantum cluster transformations. Thus, the action $\rho (S)$ on $L^2_{\textrm{sym}}(\mathbb {R}^n, m({\varvec{\lambda }})d{\varvec{\lambda }})$ can be defined as

$$\begin{aligned} \rho (S) = \mathcal {W}I_S \mathcal {W}^*, \end{aligned}$$

where $I_S$ denotes the action of *S* on $L^2(\mathbb {R}^n, d{\varvec{x}})$, see (2.16). Expressing the eigenfunctions of Macdonald operators as

$$\begin{aligned} m({\varvec{\lambda }})^{-1} \rho (S) \delta ({\varvec{\lambda }}-{\varvec{\mu }}), \end{aligned}$$

where $m(\lambda )^{-1}$ is the normalization constant, and using the fact that

$$\begin{aligned} \mathcal {W}^* \delta ({\varvec{\lambda }}-{\varvec{\mu }}) = \Psi _{\varvec{\mu }}({\varvec{x}}), \end{aligned}$$

we see that the matrix coefficients $\left\langle \Psi _\lambda , I_S \Psi _\mu \right\rangle$ give an eigenbasis for the Macdonald operators.2.16After reviewing the general construction of quantum cluster varieties in Sect. 3, the quantum cluster algebra needed to describe the spherical DAHA will be obtained from the moduli space of framed *GL*(2)-local systems on the punctured torus in Sect. 4. In Sect. 5, we use the matrix coefficient presentation to rederive the formula for the Ruijsenaars wavefunctions along with some of its properties. Finally, in Sect. 6 we recover Macdonald polynomials as special values of analytically continued Hallnäs–Ruijsenaars wavefunctions evaluated on an integer lattice. In a similar way, we recover *q*-Whittaker polynomials from *q*-Whittaker functions, as well as the corresponding Harish–Chandra series.

## Quantum cluster varieties

In this section, we review the definition of quantum cluster varieties and their representations. For more details on these subjects, we refer the reader to the foundational paper [13]. We only need skew-symmetric quantum cluster algebras with integer-valued forms, which we incorporate in the definition of a seed.

### Definition 3.1

A *seed* is a datum $\Theta =\left( I, I_0, \Lambda , (\cdot ,\cdot ),\left\{ e_i\right\} \right)$ where

- *I* is a finite set;
- $I_0 \subset I$ is a *frozen* subset of *I*;
- $$\Lambda $$ is a lattice;
- $(\cdot ,\cdot )$ is a skew-symmetric $\mathbb {Z}$-valued form on $\Lambda$;
- $\left\{ e_i \,|\, i \in I\right\}$ is a basis for the lattice $\Lambda$.

Note that the data of the last point are equivalent to that of an isomorphism ${\varvec{e}} :\mathbb {Z}^{I} \simeq \Lambda$. In particular, given a pair of seeds $(\Theta ,\Theta ')$ with the same data $\left( I,I_0\right)$, we get a canonical isomorphism of abelian groups (not necessarily isometry of lattices) $\varvec{e'} {\varvec{e}}^{-1} :\Lambda \simeq \Lambda '$.

### Definition 3.2

We say that $(\Theta ,\Theta ')$ are *equivalent* if the isomorphism $\varvec{e'} {\varvec{e}}^{-1} :\Lambda \simeq \Lambda '$ is an isometry, that is, $(e_i,e_j)_{\Lambda } = (e'_i, e'_j)_{\Lambda '}$ for all $i,j \in I$. We define a *quiver* to be an equivalence class of seeds.

The quiver *Q* associated with a seed $\Theta$ can be visualized as a directed graph with vertices labeled by the set *I* and arrows given by the adjacency matrix $\varepsilon = \left( \varepsilon _{ij}\right)$, where $\varepsilon _{ij} = (e_i,e_j)$. The vertices corresponding to the subset $I_0$ are called *frozen* and are drawn as squares, while those corresponding to $I {\setminus } I_0$ are referred to as *mutable* and are drawn as circles.

The pair $\left( \Lambda ,(\cdot , \cdot )\right)$ determines a *quantum torus algebra* ${\mathcal {T}}_\Lambda ^q$, which is defined to be the free $\mathbb {Z}[q^{\pm 1}]$-module spanned by $\left\{ Y_\lambda \,|\, \lambda \in \Lambda \right\}$, with the multiplication defined by

$$\begin{aligned} q^{(\lambda ,\mu )}Y_\lambda Y_\mu = Y_{\lambda +\mu }. \end{aligned}$$

A basis $\left\{ e_i\right\}$ of the lattice $\Lambda$ gives rise to a distinguished system of generators for ${\mathcal {T}}_\Lambda ^q$, namely the elements $Y_i=Y_{e_i}$. This way we obtain a *quantum cluster* $\mathcal {X}$-*chart*

3.1 $$\begin{aligned} \mathcal {T}_Q^q = \mathbb {Z}[q^{\pm 1}]\left\langle Y_i^{\pm 1} \,|\, i \in I\right\rangle / \left\langle q^{\varepsilon _{jk}}Y_jY_k = q^{\varepsilon _{kj}}Y_kY_j\right\rangle \simeq \mathcal {T}_\Lambda ^q. \end{aligned}$$

The generators $Y_i$ are the *quantum cluster* $\mathcal {X}$-*variables*. We note that this presentation of $\mathcal {T}_Q^q$ depends only on the quiver and not on the choice of the representative seed.

A seed also determines a Heisenberg algebra

$$\begin{aligned} \mathfrak {heis}_\Lambda =\Lambda \otimes _\mathbb {Z}\mathbb {R}\oplus \mathbb {R}c \end{aligned}$$

with generators $\left\{ y_\lambda \,|\, \lambda \in \Lambda \right\}$ and *c*, Lie bracket

$$\begin{aligned} {[}y_\lambda ,y_\mu ]=\left( \lambda ,\mu \right) c, \end{aligned}$$

and the $*$-structure

$$\begin{aligned} *y_\lambda =y_\lambda , \qquad *c=-c. \end{aligned}$$

If the form $\left( \cdot ,\cdot \right)$ is non-degenerate, the Lie algebra $\mathfrak {heis}_\Lambda$ has an irreducible $*$-representation $\rho$ on a Hilbert space ${\mathcal {H}}$ in which the generators $y_\lambda$ act by unbounded, essentially self-adjoint operators, and the central element *c* acts by the scalar $1/2\pi i$ with $i = \sqrt{-1}$. We write $\textrm{Heis}_{\Lambda }$ for the quotient of the universal enveloping algebra $U(\mathfrak {heis}_\Lambda )$ by the ideal $\left\langle 2\pi i c-1\right\rangle$.

The assignment

$$\begin{aligned} Y_\lambda = e^{2\pi \hbar y_\lambda } \end{aligned}$$

defines an embedding of ${\mathcal {T}}^q_Q$ into the set of grouplike elements in $\textrm{Heis}^\hbar _{\Lambda }$, the central quotient of the $\hbar$-adically completed universal enveloping algebra $U(\mathfrak {heis}_\Lambda )$. In the Hilbert space representation ${\mathcal {H}}$, the quantum torus generators $Y_{\lambda }$ act by unbounded, positive, essentially self-adjoint operators.

If $\Theta ,\Theta '$ are two seeds with non-degenerate skew forms representing the same quiver, then the isometry $\Lambda \rightarrow \Lambda '$, $e_i\mapsto e_i'$ determines a canonical isomorphism of Heisenberg algebras $\iota :\mathfrak {heis}_\Lambda \rightarrow \mathfrak {heis}_{\Lambda '}$. So by the irreducibility of the canonical representations ${\mathcal {H}},{\mathcal {H}}'$, there is a *unique* projective unitary equivalence ${\mathbb {I}} :\mathcal {H}\rightarrow \mathcal {H}'$ such that

3.2 $$\begin{aligned} \rho '(\iota (a))\circ {\mathbb {I}}= {\mathbb {I}} \circ \rho (a) \qquad {\text {for all}}\qquad a\in \mathfrak {heis}_\Lambda , \end{aligned}$$

where both sides are understood as maps between the corresponding classical Schwartz spaces in ${\mathcal {H}}_\Theta ,{\mathcal {H}}_{\Theta '}$. This shows that the data $(\Lambda ,\mathfrak {heis}_\Lambda ,\mathcal {H})$ associated with a non-degenerate quiver is unique up to unique isomorphism. Because of this, we will often abuse language and speak of “the” Heisenberg algebra, or Hilbert space, associated with a given quiver.

When the form $\left( \cdot ,\cdot \right)$ has nonzero kernel *Z*, the Heisenberg algebra has a family of irreducible $*$-representations ${\mathcal {H}}_{\chi }$ parametrized by central characters $\chi :Z \rightarrow \mathbb {R}$. In this case, we need to enrich the data of a quiver in order to unambiguously speak of its Heisenberg algebra and representation as explained above. Indeed, unless the central characters are compatible, the isomorphism in (3.2) cannot exist.

### Definition 3.3

A *seed with central character* is a pair $(\Theta ,\chi )$ where $\Theta$ is a seed and $\chi :Z\rightarrow \mathbb {R}$ is a linear functional on the kernel *Z* of the corresponding skew-form. We say that two seeds $(\Theta ,\chi )$ and $(\Theta ',\chi ')$ with central character are equivalent if the underlying seeds are equivalent, and the canonical isometry $\iota :\Lambda \rightarrow \Lambda '$ of lattices intertwines the central characters: $\left( \chi '\circ \iota \right) |_Z = \chi$. A *quiver with central character* is an equivalence class of seeds with central character.

Let $\Theta ,\Theta '$ be seeds representing quivers $Q,Q'$ (possibly with central characters). We say that the quiver $Q'$ is the *mutation of* *Q* *in direction* $k\in I{\setminus } I_0$ if the map

3.3 $$\begin{aligned} \mu _k :\Lambda \longrightarrow \Lambda ', \qquad e_i \longmapsto {\left\{ \begin{array}{ll} -e'_k & {\text {if}} \; i=k, \\ e'_i + \max \{(e_i,e_k),0\}e'_k & {\text {if}} \; i \ne k \end{array}\right. } \end{aligned}$$

is an isometry intertwining the central characters. It is easy to see that $Q'=\mu _k(Q)$ if and only if $Q=\mu _k(Q')$. The *mutation class* of a quiver *Q*, which we denote by the bold symbol ${\varvec{Q}}$, is the set of all quivers related to *Q* by some finite sequence of mutations.

To each quiver mutation $\mu _k$, we associate an isomorphism of quantum tori

$$\begin{aligned} \mu '_k :\mathcal {T}_Q^q \longrightarrow \mathcal {T}_{\mu _k(Q)}^q \end{aligned}$$

and define the *quantum cluster* $\mathcal {X}$-*mutation*

$$\begin{aligned} \mu ^q_k :\operatorname {Frac}(\mathcal {T}_{Q}^q) \longrightarrow \operatorname {Frac}(\mathcal {T}_{Q'}^q), \qquad f \longmapsto \Psi _q\left( Y_k'\right) \mu '_k(f) \Psi _q\left( Y_k'\right) ^{-1} \end{aligned}$$

where $\operatorname {Frac}(\mathcal {T}_Q)$ denotes the skew fraction field of the Ore domain $\mathcal {T}_Q$, and $\Psi _q(Y)$ is the (compact) quantum dilogarithm function (A.17). The fact that conjugation by $\Psi _q\left( Y'_{k}\right)$ yields a birational automorphism is guaranteed by the integrality of the form $(\cdot , \cdot )$ and the functional equations (A.18).

### Definition 3.4

An element of $\mathcal {T}^q_Q$ is said to be *universally Laurent* if its image under any finite sequence of quantum cluster mutations is contained in the corresponding quantum torus algebra. The *universally Laurent algebra* $\mathbb {L}^q_{{\varvec{Q}}}$ is the algebra of universally Laurent elements of $\mathcal {T}^q_Q$.

If *q* is a complex number, we can also consider the ${\mathbb {C}}$-algebra given by the corresponding specialization of $\mathbb {L}^q_{{\varvec{Q}}}$. We abuse notation and denote this algebra by the same symbol.

### Definition 3.5

Let $b\in {\mathbb {R}}_{>0}$ and consider the specializations

$$\begin{aligned} \hbar = b, \quad q = e^{\pi ib^2},\quad {{\tilde{q}}} = e^{\pi ib^{-2}}, \end{aligned}$$

so that $q,{{\tilde{q}}}$ are complex numbers on the unit circle. The algebra

$$\begin{aligned} \mathbb {L}_{{\varvec{Q}}} = \mathbb {L}^{q, {{\tilde{q}}}}_{{\varvec{Q}}} = \mathbb {L}^q_{{\varvec{Q}}} \otimes _{{\mathbb {C}}} \mathbb {L}^{{{\tilde{q}}}}_{{\varvec{Q}}} \end{aligned}$$

is called the *modular double* of the universally Laurent algebra $\mathbb {L}^q_{{\varvec{Q}}}$. The maximal joint domain ${\mathcal {S}}_Q\subset {\mathcal {H}}_Q$ for the action of $\mathbb {L}_{{\varvec{Q}}}$ is called the *Fock–Goncharov Schwartz space*.

### Remark 3.6

In a more general setup, when the bilinear form $(\cdot ,\cdot )$ is not required to be integer-valued, the cross-relations between elements of $\mathcal {T}^q_Q$ and $\mathcal {T}^{{{\tilde{q}}}}_Q$ are set to be

$$\begin{aligned} Y_j {{\widetilde{Y}}}_k = e^{2 \pi i \varepsilon _{kj}} {{\widetilde{Y}}}_k Y_j. \end{aligned}$$

The collection of quantum charts $\mathcal {T}^q_Q$ with $Q \in {\varvec{Q}}$, together with quantum cluster $\mathcal {X}$-mutations is often referred to as the *quantum cluster* $\mathcal {X}$-*variety*. We regard the quantum charts as the quantized algebras of functions on the toric charts in the atlas for the classical cluster Poisson variety. The quantum charts form an $\ell$-regular tree with $\ell = |I {\setminus } I_0|$, and the cluster mutations quantize the gluing data between adjacent charts. The universally Laurent algebra is the quantum analog of the algebra of global functions on the cluster variety. Unless otherwise specified in what follows, we will simply write “cluster variety” for quantum cluster $\mathcal {X}$-variety—the same applies to variables, charts, mutations, etc.

The *(quasi-)cluster modular groupoid* associated with a cluster variety is defined as follows.

### Definition 3.7

Let $Q,Q'$ be two quivers with the same label sets $(I,I_0)$. We define a *generalized permutation* to be a monomial isomorphism of quantum tori $\vartheta :\mathcal {T}_{Q}\rightarrow \mathcal {T}_{Q'}$ such that for some permutation $\sigma :(I{\setminus } I_0)\rightarrow (I{\setminus } I_0)$ of the non-frozen directions we have $\vartheta (Y_i) = Y'_{\sigma (i)}$ for all $i\in I{\setminus } I_0$. If the quivers $Q,Q'$ are equipped with central characters, we require these to be intertwined by the monomial map $\vartheta$.

In particular, the non-frozen submatrices of the matrices $\varepsilon$, $\varepsilon '$ for quivers *Q*, $Q'$ related by a generalized permutation $\vartheta$ are conjugate under an actual permutation $\sigma$ of the non-frozen set $I{\setminus } I_0$.

By the same construction as (3.2) with $\vartheta$ playing the role of $\iota$, we associate with each generalized permutation $\vartheta$ a projective unitary transformation ${\mathbb {I}}_\vartheta :{\mathcal {H}}_Q\longrightarrow {\mathcal {H}}_{Q'}.$

### Definition 3.8

Let $Q,Q'$ be two quivers with corresponding quantum tori $\mathcal {T}_Q,\mathcal {T}_{Q'}$ as in (3.1). A *quasi-cluster transformation* with source *Q* and target $Q'$ is a non-commutative birational isomorphism $\mathcal {T}_Q\dashrightarrow \mathcal {T}_{Q'}$ which can be factored as a composition of cluster mutations and generalized permutations.

### Definition 3.9

The *quasi-cluster modular groupoid* is the groupoid $\mathcal {G}_{\varvec{Q}}$ whose objects are quivers $Q \in {\varvec{Q}}$ (possibly with central characters), and whose morphisms are quasi-cluster transformations. The *quasi-cluster modular group*, denoted $\Gamma _{{\varvec{Q}}}$, is the automorphism group of an object in $\mathcal {G}_{{\varvec{Q}}}$.

### Remark 3.10

Any element of the quasi-cluster modular group restricts to an automorphism of the universally Laurent algebra $\mathbb {L}_{{\varvec{Q}}}$.

Denote by $\textbf{Hilb}$ the category whose objects are Hilbert spaces and whose morphisms are projective unitary transformations. At this point, we have associated with each object *Q* in the groupoid $\mathcal {G}_{{\varvec{Q}}}$ a Hilbert space ${\mathcal {H}}_Q$, and associated unitary intertwiners ${\mathbb {I}}_\vartheta$ to those morphisms in $\mathcal {G}_{{\varvec{Q}}}$ given by generalized permutations $\vartheta$. In [13], it was further shown how to associate intertwiners ${\mathbb {I}}_{\mu _k}$ to the mutation morphisms in $\mathcal {G}_{{\varvec{Q}}}$, which send ${\mathcal {S}}_Q$ to ${\mathcal {S}}_{Q'}$ and give rise to well-defined functor ${\mathbb {I}} :\mathcal {G}_{{\varvec{Q}}}\rightarrow \textbf{Hilb}$, that is, a *projective representation* of the groupoid $\mathcal {G}_{{\varvec{Q}}}$.

Let us briefly recall the construction of the intertwiner corresponding to a mutation $\mu _k :Q\rightarrow Q'$. Once again, the construction  (3.2) with the monomial isomorphism $\mu _k'$ playing the role of $\iota$ provides a projective unitary transformation ${\mathbb {I}}_{\mu _k'} :{\mathcal {H}}_Q\rightarrow {\mathcal {H}}_{Q'}.$ On the other hand, by the property (A.1) of the quantum dilogarithm function $\varphi (z)$ it follows that any self-adjoint operator *y* on ${\mathcal {H}}$ defines a unitary operator $\varphi (y) :{\mathcal {H}}\simeq {\mathcal {H}}$. The unitary transformation ${\mathbb {I}}_{\mu _k} :{\mathcal {H}}_Q \rightarrow {\mathcal {H}}_{Q'}$ is then defined to be

3.4 $$\begin{aligned} {\mathbb {I}}_{\mu _k} = {\mathbb {I}}_{\mu _k'}\circ \varphi (-y_k)^{-1}=\varphi (y'_k)^{-1}\circ {\mathbb {I}}_{\mu _k'}. \end{aligned}$$

That this recipe is indeed well defined is a difficult theorem, which is the main analytical result of the paper [13]. In the following section, we will illustrate how to use it in practice with a concrete example.

## Quantum Teichmüller theory for the punctured torus

In this section, we illustrate the previous definitions in our main example, the moduli space of framed $GL_2$ local systems on a punctured torus.

### Quantized moduli space of framed $GL_2$-local systems on a punctured torus

**Fig. 2:**
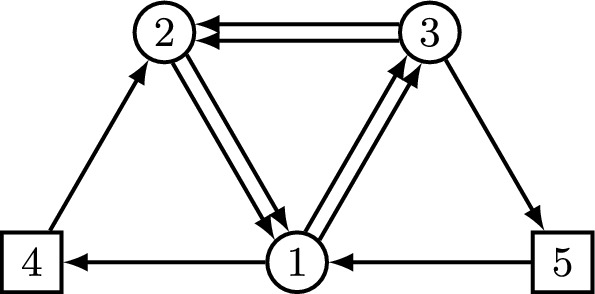
Quiver *Q*

Consider the quiver *Q* shown in Fig. 2 and the associated cluster variety. The kernel of the corresponding skew-form is spanned by the vector $z = -e_1-e_2-e_3$. For $\tau \in {\mathbb {R}}$, we write $Q_\tau$ for the quiver with central character defined by ${\mathcal {X}}_\tau (z)={2}\tau$. The latter corresponds to a relation

$$\begin{aligned} Y_1Y_2Y_3 = q^2e^{-4\pi b \tau }. \end{aligned}$$

The mutable part of the quiver is known as the *Markov quiver* and is isomorphic to any of its mutations. The Markov quiver describes the cluster structure on the moduli space of framed $SL_2$-local systems on a punctured torus, where the framing data consist of a flag (a line in rank 1), invariant under the monodromy around the puncture, see [12]. The additional frozen vertices will be used to parametrize the determinants of holonomies around loops on the punctured torus, as we now explain.

**Fig. 3:**
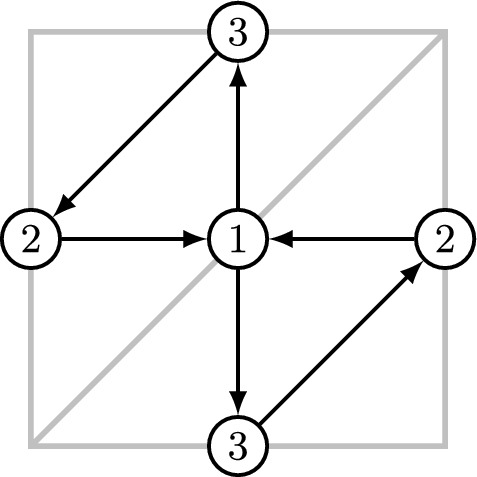
Cluster quiver from a punctured torus

At the quantum level, the algebra $\mathbb {L}_{{\varvec{Q}}}$ contains elements corresponding to the traces and determinants of holonomies around closed simple curves. For $(m,n) \in \mathbb {Z}^2$, we denote by $L_{(m,n)}$ and $\Delta _{(m,n)}$ the elements corresponding, respectively, to the trace and the determinant of the monodromy along the (*m*, *n*)-curve on the punctured torus. These elements can be constructed explicitly as follows. We choose a basis in $H_1(T^2{\setminus } D^2;\mathbb {Z})\simeq {\mathbb {Z}}^2$ so that the horizontal cycle, passing from left to right through nodes 1 and 2 in Fig. 3, is of homology class (1, 0). Then, we set

4.1 $$\begin{aligned} L_{(1,0)} = Y_{e_4} + Y_{e_2+e_4} + Y_{e_1+e_2+e_4}, \qquad \Delta _{(1,0)} = Y_{e_1+e_2+2e_4}. \end{aligned}$$

As shown in [29, Proposition 3.4], elements $L_{(1,0)}$ and $\Delta _{(1,0)}$ are universally Laurent:

$$\begin{aligned} L_{(1,0)}, ~\Delta _{(1,0)} \in \mathbb {L}_{{\varvec{Q}}}. \end{aligned}$$

**Fig. 4:**
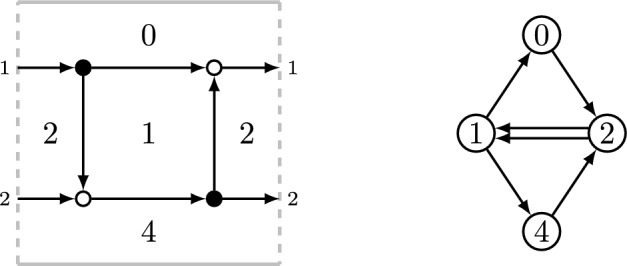
Directed network and dual cluster quiver

Formulas (4.1) can be written down with the help of the following standard combinatorial recipe, see, e.g., [12, Section 9]. Consider the bipartite graph on a cylinder, shown in the left of Fig. 4. On the right, we show a dual quiver, which we use to define a cluster variety, with edges directed in such a way that the white vertex of the bipartite graph stays on the right as we traverse an edge. The direction of edges of the bipartite graph is additional data, which allows one to express a monodromy matrix *M* in cluster coordinates. Namely, we set

$$\begin{aligned} M_{ij} = \sum _{p: \, i \rightarrow j} Y_{\operatorname {wt}(p)}, \qquad {\text {where}}\qquad \operatorname {wt}(p) = \sum _{f \, {\text {below}} \, p} e_f. \end{aligned}$$

The first sum in the formula above is taken over all paths in the directed bipartite graph from *i*-th source to the *j*-th sink, while the second is taken over all faces lying below the path $\operatorname {wt}(p)$. Note that the matrix *M* only depends on variables 1, 2, and 4, but not on the variable 0. Since the monodromy matrix is only defined up to conjugation, we shall be looking at the symmetric functions of its eigenvalues: the trace and the determinant in our case, which are equal to $L_{1,0}$ and $\Delta _{1,0}$, respectively. Let us also note that in order to work with $SL_2$ rather than $GL_2$ local systems one only needs to specialize $y_4 = -\frac{1}{2}(y_1+y_2)$.

The mapping class group of a punctured torus is isomorphic to $SL(2,\mathbb {Z})$ and is generated by the elements

$$\begin{aligned} \sigma _+ = \begin{pmatrix} 1 &  1 \\ 0 &  1 \end{pmatrix} \qquad {\text {and}}\qquad \sigma _- = \begin{pmatrix} 1 &  0 \\ 1 &  1 \end{pmatrix} \end{aligned}$$

which correspond to the Dehn twists of the torus along closed simple curves with homology classes (1, 0) and (0, 1), respectively. By the construction in [13, Section 6], we get a homomorphism $SL(2,\mathbb {Z}) \rightarrow \Gamma _{{\varvec{Q}}_\tau }$ sending

4.2 $$\begin{aligned} \sigma _+^{-1} \longmapsto m_+ \circ \mu _1^q, \qquad \sigma _- \longmapsto m_- \circ \mu _3^q, \end{aligned}$$

where $m_+ :{\mathcal {T}}_{\mu _1(Q_\tau )} \rightarrow {\mathcal {T}}_{Q_\tau }$ and $m_- :{\mathcal {T}}_{\mu _3(Q_\tau )} \rightarrow {\mathcal {T}}_{Q_\tau }$ are the following generalized permutations:

4.3 $$\begin{aligned} \begin{aligned} m_+&= \left\{ e'_1 \mapsto e_2, \, e'_2 \mapsto e_1, \, e'_3 \mapsto e_3, \, e'_4 \mapsto e_4, \, e'_5 \mapsto e_1+e_4+e_5\right\} ,  &   e'_j = \mu _1(e_j), \\ m_-&= \left\{ e'_1 \mapsto e_3, \, e'_2 \mapsto e_2, \, e'_3 \mapsto e_1, \, e'_4 \mapsto -e_1-e_3+e_4-e_5, \, e'_5 \mapsto e_5\right\} ,  &   e'_j = \mu _3(e_j). \end{aligned}\nonumber \\ \end{aligned}$$

The element $\sigma$ of order 6 defined by

$$\begin{aligned} \sigma = \sigma _+^{-1}\sigma _- = \begin{pmatrix} 0 &  -1 \\ 1 &  1 \end{pmatrix} \end{aligned}$$

is mapped under this homomorphism to a generalized permutation:

$$\begin{aligned} \sigma \longmapsto \left\{ e_1 \mapsto e_3, \, e_2 \mapsto e_1, \, e_3 \mapsto e_2, \, e_4 \mapsto -e_1-e_3-e_5, \, e_5 \mapsto e_1+e_4+e_5\right\} . \end{aligned}$$

Most important for us will be the order 4 element

$$\begin{aligned} S = \sigma _+^{-1}\sigma _-\sigma _+^{-1} = \begin{pmatrix} 0 &  -1 \\ 1 &  0 \end{pmatrix}. \end{aligned}$$

Given $$g = \begin{pmatrix} a &  b \\ c &  d \end{pmatrix} \in SL(2,\mathbb {Z})$$ we define

4.4 $$\begin{aligned} L_{(a,c)} = g\cdot L_{(1,0)}, \qquad \Delta _{(a,c)} = g\cdot \Delta _{(1,0)}. \end{aligned}$$

The above definitions make sense thanks to the fact that

$$\begin{aligned} \sigma _+\left( L_{(1,0)}\right) = L_{(1,0)} \qquad {\text {and}}\qquad \sigma _+\left( \Delta _{(1,0)}\right) = \Delta _{(1,0)}. \end{aligned}$$

For example, the element *S* satisfies

4.5 $$\begin{aligned} S\left( L_{(1,0)}\right) = L_{(0,1)}, \qquad S\left( \Delta _{(1,0)}\right) = \Delta _{(0,1)}. \end{aligned}$$

Note that $g L_{(1,0)}$ is universally Laurent for any $g \in SL(2,\mathbb {Z})$ since $L_{(1,0)}$ is. Using (4.4), we now compute

$$\begin{aligned} L_{(0,1)}&= Y_{e_5}^{-1} + Y_{e_3+e_5}^{-1} + Y_{e_1+e_3+e_5}^{-1},&\Delta _{(0,1)}&= Y_{e_1+e_3+2e_5}^{-1}, \end{aligned}$$

as well as

$$\begin{aligned} L_{(0,-1)}&= Y_{e_5} + Y_{e_1+e_5} + Y_{e_1+e_3+e_5},&\Delta _{(0,-1)}&= Y_{e_1+e_3+2e_5}, \\ L_{(1,-1)}&= Y_{e_1+e_4+e_5} + Y_{e_1+e_3+e_4+e_5} + Y_{e_1+e_2+e_3+e_4+e_5},&\Delta _{(1,-1)}&= Y_{2e_1+e_2+e_3+2e_4+2e_5}. \end{aligned}$$

Finally, we observe that for any $a,b,c,d \in \mathbb {Z}$, satisfying $ad-bc=1$, we have

$$\begin{aligned}  &   \Delta _{(b,d)}\Delta _{(-b,-d)} = 1, \qquad L_{(-b,-d)} = \Delta _{(b,d)}^{-1}L_{(b,d)}, \qquad \left[ L_{(a,c)},L_{(b,d)}\right] \\  &   \quad = {\left( q^{-1}-q\right) } L_{(a+c,b+d)}. \end{aligned}$$

Indeed, the above relations hold for $(a,b,c,d) = (0,1,-1,0)$, and the general case follows from (4.4).

### Unitary representation of $SL(2,{\mathbb {Z}})$

Following the construction of Sect. 3, we consider the Hilbert space ${\mathcal {H}}_Q$ associated with the quiver $Q_\tau$ from Fig. 2. In the approach to quantum Teichmüller theory of [13], the Hilbert space ${\mathcal {H}}_Q$ is the one assigned to the punctured torus.

We now compute the action of the generators $\sigma _\pm$ on ${\mathcal {H}}_Q$ under the representation (4.2). In order to write explicit formulas, let us fix the isomorphism ${\mathcal {H}}_{Q_\tau }\simeq L_2({\mathbb {R}}^2)$ such that the action of the Heisenberg generators on a Schwartz function $f(x_1,x_2)$ is given by

4.6 $$\begin{aligned} \begin{aligned}&y_1 \longmapsto p_1-p_2+x_1-x_2,  &   \qquad y_2 \longmapsto x_2-x_1,  &   \qquad y_3 \longmapsto p_2-p_1-2\tau , \\&y_4 \longmapsto p_2,  &   \qquad y_5 \longmapsto -x_1+\tau . \end{aligned} \end{aligned}$$

Under this isomorphism, the quantum holonomies act by the operators

4.7 $$\begin{aligned} \begin{aligned} L_{(1,0)}&\longmapsto e^{2\pi b p_2} + e^{2\pi b(p_2+x_2-x_1)} + e^{2\pi bp_1},&\qquad \Delta _{(1,0)}&\longmapsto e^{2\pi b(p_1+p_2)}, \\ L_{(0,1)}&\longmapsto e^{2\pi b(x_1-\tau )} + e^{2\pi b(p_1-p_2+x_1+\tau )} + e^{2\pi b(x_2+\tau )},&\qquad \Delta _{(0,1)}&\longmapsto e^{2\pi b(x_1+x_2)}. \end{aligned} \end{aligned}$$

Note that $L_{(1,0)}$ and $\Delta _{(1,0)}$ are mapped, respectively, to the Toda Hamiltonians $H_1$ and $H_2$ defined in (2.6). It is then easy to check that the projective transformations ${\mathbb {I}}_{\sigma _\pm } :{\mathcal {H}}_{Q_\tau }\rightarrow {\mathcal {H}}_{Q_\tau }$ take the following form:

4.8 $$\begin{aligned} \begin{aligned} {\mathbb {I}}_{\sigma _+^{-1}}&= e^{-\pi i(p_1^2+p_2^2)} \varphi (p_2-p_1+x_2-x_1)^{-1},\\ {\mathbb {I}}_{\sigma _-}&= e^{2\pi i \tau (x_1-x_2)} e^{-\pi i(x_1^2+x_2^2)} \varphi (p_1-p_2+2\tau )^{-1}. \end{aligned} \end{aligned}$$

#### Remark 4.1

Comparing formulas (2.8) and (4.8), we see that the *Q*-system evolution operator is identified with the action $I_{\sigma _+}$ of the (1, 0)-cycle Dehn twist, justifying our notation for the former.

To illustrate the construction, we spell out the derivation of the projective transformation $\sigma _+^{-1}$. Composing the maps (3.3) and (4.3), we find that the monomial automorphism $m_+\circ \mu _1'$ of $\mathfrak {heis}_{Q_\tau }$ is given by

$$\begin{aligned} \begin{aligned}&y_1 \longmapsto -y_2,  &   \qquad y_2 \longmapsto y_1+2y_2,  &   \qquad y_3 \longmapsto y_3, \\&y_4 \longmapsto y_4,  &   \qquad y_5 \longmapsto y_1+y_2+y_4+y_5. \end{aligned} \end{aligned}$$

The monomial part ${\mathbb {I}}_{m_+}\circ {\mathbb {I}}_{\mu _1'}$ of the intertwiner ${\mathbb {I}}_{\sigma _+^{-1}}$ is then defined as the unique projective transformation satisfying the intertwining relations

$$\begin{aligned} ({\mathbb {I}}_{m_+}\circ {\mathbb {I}}_{\mu _1'})\circ y_k = (m_+\circ \mu _1')(y_k)\circ ({\mathbb {I}}_{m_+}\circ {\mathbb {I}}_{\mu _1'}) \qquad {\text {for all}}\qquad 1\leqslant k\leqslant 5. \end{aligned}$$

It is straightforward to check that under the isomorphism (4.6), these relations are satisfied by the operator $e^{-\pi i(p_1^2+p_2^2)}$. The formula for $\sigma _-$ is obtained in the same way.

Rewriting the latter expression as

$$\begin{aligned} {\mathbb {I}}_{\sigma _-} = \varphi (p_1-p_2+x_1-x_2)^{-1} e^{2\pi i \tau (x_1-x_2)} e^{-\pi i(x_1^2+x_2^2)}, \end{aligned}$$

we may use the inversion formula (A.6) to see that the element $\sigma$ is mapped to the projective transformation

4.9 $$\begin{aligned} {\mathbb {I}}_{\sigma } \longmapsto e^{2\pi ix_1x_2} \mathcal {F}e^{2\pi i\tau (x_1-x_2)}. \end{aligned}$$

Here, $\mathcal {F}$ is the Fourier transform that may be written as

$$\begin{aligned} \mathcal {F}:f({\varvec{x}}) \longmapsto \int _{\mathbb {R}^2} f({\varvec{y}}) e^{-2\pi i \varvec{x} \cdot {\varvec{y}}} d{\varvec{y}}, \end{aligned}$$

or, see, e.g., [32], in an equivalent form

$$\begin{aligned} \mathcal {F}= i e^{-\pi i (p_1^2+p_2^2)} e^{-\pi i (x_1^2+x_2^2)} e^{-\pi i (p_1^2+p_2^2)}. \end{aligned}$$

In fact, choosing the following lifts of the projective transformations ${\mathbb {I}}_{\sigma _+^{-1}}, {\mathbb {I}}_{\sigma _-}$ to linear ones defines a lift of the projective representation ${\mathbb {I}}$ to a unitary representation *I* of the same group $SL(2,{\mathbb {Z}})$:

4.10 $$\begin{aligned} \begin{aligned} {I}_{\sigma _+^{-1}}&= \zeta _se^{\frac{2}{3}\pi i \tau ^2}e^{-\pi i(p_1^2+p_2^2)} \varphi (p_2-p_1+x_2-x_1)^{-1},\\ {I}_{\sigma _-}&= \zeta _s^{-1}e^{-\frac{4}{3}\pi i \tau ^2}e^{2\pi i \tau (x_1-x_2)} e^{-\pi i(x_1^2+x_2^2)} \varphi (p_1-p_2+2\tau )^{-1}, \end{aligned} \end{aligned}$$

where we have set

4.11 $$\begin{aligned} \zeta _s = e^{{\frac{\pi i}{6}(1-c_b^2) }}. \end{aligned}$$

Most important for us will be the unitary automorphism $I_S$ of $L^2(\mathbb {R}^2,d{\varvec{x}})$:

4.12 $$\begin{aligned} I_S = I_{\sigma _+^{-1}}I_{\sigma _-}I_{\sigma _+^{-1}}. \end{aligned}$$

#### Proposition 4.2

The element $I_S^2$ acts on $L^2(\mathbb {R}^2,d{\varvec{x}})$ by

$$\begin{aligned} (I_S^2f)({\varvec{x}}) = f({\varvec{x}}^*) \qquad {\text {where}}\qquad {\varvec{x}}^* = (-x_2,-x_1). \end{aligned}$$

In particular, $I_S$ is a unitary automorphism of $L^2(\mathbb {R}^2,d{\varvec{x}})$ of order 4.

#### Proof

The proof consists of a direct verification based on the definitions (4.10) and (4.12), the inversion formula (A.6), the factorization

$$\begin{aligned} \mathcal {F}= i e^{-\pi i (p_1^2+p_2^2)} e^{-\pi i (x_1^2+x_2^2)} e^{-\pi i (p_1^2+p_2^2)}, \end{aligned}$$

and the relation

$$\begin{aligned} (e^{2\pi i p_1p_2}e^{2\pi i x_1x_2}e^{2\pi i p_1p_2}f)({\varvec{x}}) = (\mathcal {F}f)({\varvec{x}}^*). \end{aligned}$$

$$\square $$

### Cluster realization of spherical double affine Hecke algebra

We now algebraically interpret the computations from the previous section as defining an $SL(2;{\mathbb {Z}})$-equivariant injective homomorphism from the spherical subalgebra ${\mathbb{S}\mathbb{H}}_{q,t}$ of the ${\mathfrak {gl}}_2$ double affine Hecke algebra $\mathbb {H}_{q,t}$ into the universally Laurent algebra $\mathbb {L}^q_{{\varvec{Q}}}$. The ${\mathfrak {gl}}_n$ version of this homomorphism will be given in a forthcoming publication.

#### Theorem 4.3

There is an $SL(2,\mathbb {Z})$-equivariant injective homomorphism

$$\begin{aligned} \iota :{\mathbb{S}\mathbb{H}}_{q,t} \hookrightarrow \mathbb {L}^q_{{\varvec{Q}}}, \end{aligned}$$

defined by

$$\begin{aligned} \iota \left( E_{(\pm 1,0)}\right) = L_{(\pm 1,0)} \qquad {\text {and}}\qquad \iota \left( E_{(0,\pm 1)}\right) = L_{(0,\pm 1)}. \end{aligned}$$

#### Proof

Comparing formulas (2.9) and (2.15) with the ones  (4.7) derived in the previous section shows that for each $(a,b)\in \{(\pm 1,0),(0,\pm 1)\}$, the inverse Whittaker transform

$$\begin{aligned} \mathcal {W}^* :L^2_{\textrm{sym}}(\mathbb {R}^2, m({\varvec{\lambda }})d{\varvec{\lambda }}) \longrightarrow L^2(\mathbb {R}^2, d{\varvec{x}}) \end{aligned}$$

intertwines the action of the spherical DAHA generator $E_{(a,b)}$ in the Cherednik representation with that of the quantum cluster algebra element $L_{(a,b)}$ in the representation ${\mathcal {S}}_\tau \subset {\mathcal {H}}_\tau$ of the quantum cluster variety. Hence, Theorem 2.4, together with the faithfulness of the two representations at hand, guarantees that $\iota$ is an injective homomorphism. The $SL(2,\mathbb {Z})$-equivariance is manifest, since for any $g \in SL(2,{\mathbb {Z}})$ and any primitive vector $v \in \mathbb {Z}^{ 2}$ we have $L_{gv} = gL_v$ and $E_{gv} = gE_v$. $\square$

We therefore see that in the context of quantum Teichmüller theory for the punctured torus as formulated in Sect. 4, the Whittaker functions $\Psi _{\varvec{\lambda }}({\varvec{x}})$ are interpreted as eigenfunctions for the quantized traces of monodromy around the (1, 0)-curve. Equation  (4.5) is interpreted as the intertwining relation

4.13 $$\begin{aligned} M^{\tau }_j \mathcal {W}I_S = \mathcal {W}I_S H_j. \end{aligned}$$

between the Toda and Macdonald operators under the unitary equivalence

$$\begin{aligned} \mathcal {W}\circ I_S :L^2(\mathbb {R}^2, d{\varvec{x}}) \longrightarrow L^2_{\textrm{sym}}(\mathbb {R}^2, m({\varvec{\lambda }})d{\varvec{\lambda }}). \end{aligned}$$

In particular, the distributions $I_S \Psi _{\varvec{\lambda }}({\varvec{x}})$ provide a complete set of eigenfunctions for the quantized traces of monodromy around the (0, 1)-curve.

## Ruijsenaars wavefunctions as matrix coefficients

In this section, we show how the eigenfunctions of Macdonald operators, the Hallnäs–Ruijsenaars functions, can be derived from those of Toda Hamiltonians using the action of $SL(2;{\mathbb {Z}})$ on the Hilbert space assigned by quantum Teichmüller theory to the punctured torus. More specifically, we present a formula for the Hallnäs–Ruijsenaars eigenfunctions as matrix coefficients between Toda eigenfunctions for the element of *S* of $SL(2,{\mathbb {Z}})$ considered in the previous section. This provides a functional version of Cherednik’s $SL(2,\mathbb {Z})$ action in the DAHA theory, see [6], where *S* acts as the *Macdonald–Fourier* integral transform with kernel being Macdonald polynomial, and $\sigma _+$ acts via multiplication by the Gaussian $\gamma$. We remark that the details on the use of the *Whittaker–Fourier* transform for the *q*-Whittaker case can be found in [10].

Recall from the previous section the eigenfunctions $I_S\cdot \Psi _{\varvec{\lambda }}({\varvec{x}})$ for the quantized traces of monodromy around the (0, 1)-curve. We first compute these eigenfunctions more explicitly.

### Proposition 5.1

The action of the unitary transformations $I_S$ and $I_S^2$ on the distribution $\Psi _{\varvec{\lambda }}({\varvec{x}})$ reads

$$\begin{aligned} I_S \Psi _{\varvec{\lambda }}({\varvec{x}})&= \zeta \zeta _s e^{-\pi i (\lambda _1^2+\lambda _2^2)} e^{\pi i c_b(2\tau -\underline{\varvec{\lambda }})} e^{2\pi i (x_1+c_b)x_2} \delta (\underline{\varvec{\lambda }}-\underline{{\varvec{x}}}) \\&\phantom { = }\varphi (x_1-\lambda _2-\tau +c_b) \varphi (\lambda _2-x_2-\tau +c_b), \\ I_S^2 \Psi _{\varvec{\lambda }}({\varvec{x}})&= \Psi _{{\varvec{\lambda }}^*}({\varvec{x}}), \end{aligned}$$

where we recall from Appendix A the phase constant $\zeta = e^{\pi i(1-4c_b^2)/12}$.

### Proof

First we observe that since $\Psi _{\varvec{\mu }}({\varvec{x}})$ is an eigenfunction of the Dehn twist $D=\sigma _+$ with eigenvalue $\gamma$ given by (2.10), we have

$$\begin{aligned} I_S \Psi _{\varvec{\mu }}({\varvec{x}}) = \zeta _s e^{-\pi i (\mu _1^2+\mu _2^2)} I_{\sigma } \Psi _{\varvec{\mu }}({\varvec{x}}). \end{aligned}$$

So it remains to calculate $I_\sigma \Psi _{\varvec{\mu }}$, which is given by the integral

$$\begin{aligned} I_\sigma \Psi _{\varvec{\mu }}({\varvec{x}}) = e^{2\pi ix_1x_2} \int e^{-2 \pi i(x_1y_1+x_2y_2)} e^{2\pi i \tau (y_1-y_2)} \Psi _{\varvec{\mu }}({\varvec{y}}) d\varvec{y}. \end{aligned}$$

Using the Gauss–Givental formula (2.11) and substituting $y_1 = s-z_1$, $y_2 = z_2+s-c_b$, we arrive at

$$\begin{aligned} \zeta ^{-1} e^{-\pi i c_b \underline{\varvec{\mu }}} e^{2\pi i c_b(x_2+\tau )} \int \frac{e^{2\pi i z_1(x_1-\mu _2-\tau )} e^{2\pi i z_2(\mu _2-x_2-\tau )} e^{2\pi i s(\underline{\varvec{\mu }}-\underline{\varvec{x}})}}{\varphi (z_1-c_b)\varphi (z_2-c_b)} ds d{\varvec{z}}. \end{aligned}$$

To finish the proof of the first formula, we take the three integrals in the above expression by applying the standard formula for the delta function

$$\begin{aligned} \int e^{2 \pi i s x} ds = \delta (x) \end{aligned}$$

along with the quantum dilogarithm Fourier transform (A.10).

In view of Proposition 4.2, the second formula for the action of $I_S^2$ follows from comparing the Gauss–Givental presentation (2.11) for $\Psi _{{\varvec{\lambda }}^*}({\varvec{x}})$ with the Mellin–Barnes presentation (2.12) for $\Psi _{\varvec{\lambda }}({\varvec{x}}^*)$. $\square$

We denote by $\Phi _{\varvec{\mu }}^\tau ({\varvec{\lambda }})$ the following matrix coefficient

5.1 $$\begin{aligned} \Phi _{\varvec{\mu }}^\tau ({\varvec{\lambda }}) = \left\langle \Psi _{\varvec{\lambda }}, I_S \Psi _{\varvec{\mu }}\right\rangle \end{aligned}$$

of the element $S \in SL(2,\mathbb {Z})$. The following proposition gives an explicit calculation of this matrix element. An equivalent formula has been obtained by Teschner and Vartanov in Section 6.5.4 of [33].

### Proposition 5.2

The matrix coefficient $\Phi _{\varvec{\mu }}^\tau ({\varvec{\lambda }})$ is related to the Hallnäs–Ruijsenaars eigenfunction $\textrm{P}_{\varvec{\mu }}^\tau ({\varvec{\lambda }})$ via the formula

5.2 $$\begin{aligned} \textrm{P}_{\varvec{\mu }}^\tau ({\varvec{\lambda }}) = \zeta \zeta _s^{-1} e^{2\pi i\tau ^2} \Phi _{\varvec{\mu }}^\tau ({\varvec{\lambda }}). \end{aligned}$$

In particular, the integral defining the matrix coefficient (5.1) is convergent.

### Proof

Applying (A.1) and (A.6) to the Gauss–Givental presentation (2.11) of the Whittaker function, we get

$$\begin{aligned} \overline{\Psi _{\varvec{\lambda }}({\varvec{x}})} = \zeta ^{-3} e^{-2\pi ic_b^2} e^{\pi i c_b(\lambda _2-\lambda _1)} e^{-2\pi i \lambda _2 \underline{{\varvec{x}}}} \int \frac{e^{\pi i (r-x_1+c_b)^2} e^{\pi i (x_2-r)^2} e^{2\pi i r(\lambda _2-\lambda _1)} }{\varphi (x_1-r-c_b) \varphi (r-x_2)} dr. \end{aligned}$$

We now substitute the above expression into (5.1) and apply Proposition 5.1. Integrating out $x_2$ and shifting the integration variable $x_1 = x+r$, we have

$$\begin{aligned} \left\langle \Psi _{\varvec{\lambda }}, I_S \Psi _{\varvec{\mu }}\right\rangle = \zeta _{\textrm{inv}} \zeta _s e^{\pi i c_b(\underline{\varvec{\mu }}+\lambda _2-\lambda _1+2\tau )} e^{2\pi i \mu _1\mu _2} e^{-2 \pi i \lambda _2\underline{\varvec{\mu }}} \int e^{2\pi i r(r+\lambda _2-\lambda _1-\underline{\varvec{\mu }}-c_b)} \\ e^{-4\pi i c_bx} \frac{\varphi (x+r-\mu _1-\tau +c_b) \varphi (x+r-\mu _2-\tau +c_b)}{\varphi (x+2r-\underline{\varvec{\mu }}) \varphi (x-c_b)} dx dr \end{aligned}$$

Using the identity (A.14) with $u_j = r-\mu _j-\tau +c_b$ and $v = 2r-\underline{\varvec{\mu }}+c_b$ and applying the inversion formula (A.6) three more times, we derive that the integral over *x* is equal to

$$\begin{aligned} \zeta e^{-2\pi i(\mu _1\mu _2+\underline{\varvec{\mu }}\tau +\tau ^2)} \varphi (2\tau ) e^{2\pi i r(-r+\underline{\varvec{\mu }}+2\tau )} \frac{\varphi (r-\mu _1-\tau +c_b) \varphi (r-\mu _2-\tau +c_b)}{\varphi (r-\mu _1+\tau ) \varphi (r-\mu _1-\tau )}. \end{aligned}$$

Finally, substituting $r = x + \frac{1}{2}(\underline{\varvec{\mu }}-c_b)$ we arrive at the desired formula (5.2). $\square$

The following result is an immediate consequence of Corollary 5.6 and Proposition 5.2.

### Corollary 5.3

The Hallnäs–Ruijsenaars eigenfunction $\textrm{P}_{\varvec{\mu }}^\tau ({\varvec{\lambda }})$ is symmetric in ${\varvec{\lambda }}$, ${\varvec{\mu }}$, and satisfies

$$\begin{aligned} \textrm{P}_{\varvec{\mu }}^\tau ({\varvec{\lambda }}) = \textrm{P}_{\varvec{\lambda }}^{-\tau }({\varvec{\mu }}) \qquad {\text {and}}\qquad \overline{\textrm{P}_{\varvec{\mu }}^\tau ({\varvec{\lambda }})} = \zeta _{inv}^{-1}e^{-4\pi i \tau ^2}{\textrm{P}_{\varvec{\mu }}^{-\tau }({\varvec{\lambda }}^*)}. \end{aligned}$$

### Remark 5.4

Note that the relation $\textrm{P}_{\varvec{\mu }}^\tau ({\varvec{\lambda }}) = \textrm{P}_{\varvec{\lambda }}^{-\tau }({\varvec{\mu }})$ is a composition of the *bispectral duality* $\textrm{P}_{\varvec{\mu }}^\tau ({\varvec{\lambda }}) = \textrm{P}_{\varvec{\lambda }}^{\tau }({\varvec{\mu }})$ and *Poincaré duality* $\textrm{P}_{\varvec{\mu }}^\tau ({\varvec{\lambda }}) = \textrm{P}_{\varvec{\mu }}^{-\tau }({\varvec{\lambda }})$, which hold separately.

### Theorem 5.5

Let $M^{\tau }_j$ be the *j*-th ${\mathfrak {gl}}_2$-Macdonald operator.

1. The Macdonald operators $M^{\tau }_1$, $M^{\tau }_2$ are essentially self-adjoint with respect to $\left\langle \cdot ,\cdot \right\rangle _{Sk}$.
2. The function $\Phi _{\varvec{\mu }}^\tau ({\varvec{\lambda }})$ is an eigenfunction of Macdonald operators: For $j=1,2$ one has
  $$\begin{aligned} M^{\tau }_j \Phi _{\varvec{\mu }}^\tau ({\varvec{\lambda }}) = e_j({\varvec{\mu }}) \Phi _{\varvec{\mu }}^\tau ({\varvec{\lambda }}). \end{aligned}$$
3. For any $\tau \in \mathbb {R}$, the integral transform
  $$\begin{aligned} {\mathcal {M}}:f \longmapsto \left\langle \Phi _{\varvec{\mu }}^\tau ,f\right\rangle _{Sk} \end{aligned}$$
  is a unitary automorphism of $L^2_{\textrm{sym}}(\mathbb {R}^2, m({\varvec{\lambda }})d{\varvec{\lambda }})$.

### Proof

Point 1 follows from the equality (4.13), the unitarity of $I_S$, Theorem 2.4, and the fact that Toda Hamiltonians are essentially self-adjoint with respect to $\left\langle \cdot ,\cdot \right\rangle$. Point 2 is a consequence of the equalities (2.9), (4.13). Finally, point 3 follows from the observation that the transform ${\mathcal {M}}$ is nothing but the explicit form of the unitary automorphism $\mathcal {W}I_S \mathcal {W}^*$ of $L^2_{\textrm{sym}}(\mathbb {R}^2, m({\varvec{\lambda }})d{\varvec{\lambda }})$. $\square$

### Corollary 5.6

The matrix coefficient $\Phi _{\varvec{\mu }}^\tau ({\varvec{\lambda }})$ has the following properties:

1. The function $\Phi _{\varvec{\mu }}^\tau ({\varvec{\lambda }})$ is symmetric in ${\varvec{\lambda }}$, ${\varvec{\mu }}$, and satisfies
  5.3 $$\begin{aligned} \overline{\Phi _{\varvec{\mu }}^\tau ({\varvec{\lambda }})} = \Phi _{\varvec{\mu }}^{-\tau }({\varvec{\lambda }}^*). \end{aligned}$$
2. The function $\Phi _{\varvec{\mu }}^\tau ({\varvec{\lambda }})$ satisfies the duality
  $$\begin{aligned} \Phi _{\varvec{\mu }}^\tau ({\varvec{\lambda }}) = \Phi _{\varvec{\lambda }}^{-\tau }({\varvec{\mu }}). \end{aligned}$$

### Proof

By Point 3 of Theorem 5.5, the symmetry in ${\varvec{\lambda }}$ and ${\varvec{\mu }}$ is inherited from that of the Whittaker functions, and the equality (5.3) is easily checked using the unitarity property (A.6) of the non-compact quantum dilogarithm. For Point 2, we use the symmetries in Point 1, together with the unitarity of the *S*-transformation $I_S$ and the second part of Proposition 5.1:

$$\begin{aligned} \Phi _{\varvec{\mu }}^\tau ({\varvec{\lambda }})= &   \langle \Psi _{\varvec{\lambda }},I_S \Psi _{\varvec{\mu }} \rangle \\= &   \langle I_S\Psi _{\varvec{\lambda }}, I_S^2\Psi _{\varvec{\mu }} \rangle = \langle I_S\Psi _{\varvec{\lambda }}, \Psi _{{\varvec{\mu }}^*} \rangle = \overline{ \langle \Psi _{{\varvec{\mu }}^*}, I_S\Psi _{\varvec{\lambda }} \rangle } = \overline{ \Phi _{\varvec{\lambda }}^\tau ({\varvec{\mu }}^*)} = \Phi _{\varvec{\lambda }}^{-\tau }({\varvec{\mu }}). \end{aligned}$$

$$\square $$

## Macdonald polynomials and Harish–Chandra series

We finish the paper by showing how the *GL*(2) *q*-Whittaker and Macdonald polynomials can be obtained, respectively, as special values of the analytic continuations of Whittaker and Hallnäs–Ruijsenaars functions. We also show that by closing the contour of integration and calculating residues of these functions, we recover the Harish–Chandra series in both Whittaker and Macdonald case.

### Whittaker polynomials from Whittaker functions

As observed in [28], the Whittaker function $\Psi _{\varvec{\lambda }}({\varvec{x}})$ becomes entire after multiplying by $\varphi (x_2-x_1)$. Let us define

6.1 $$\begin{aligned} {{\widetilde{\Psi }}}_{\varvec{\lambda }}({\varvec{x}}) = -\zeta ^{-1} e^{\pi i c_b (\lambda _2-\lambda _1)} e^{2\pi i (\lambda _1 x_1 + \lambda _2 x_2)} \varphi (x_2-x_1) \int _C \frac{e^{2\pi i t(\lambda _1-\lambda _2) }}{\varphi (t-c_b)\varphi (x_2-x_1-t)}dt, \end{aligned}$$

and note that ${{\widetilde{\Psi }}}_{\varvec{\lambda }}({\varvec{x}})$ is a joint eigenfunction of the mutated Hamiltonians

$$\begin{aligned} \varphi (x_2-x_1) H_1 \varphi (x_2-x_1)^{-1}&= e^{2\pi b p_1} + e^{2\pi b(p_1+x_2-x_1)} + e^{2\pi b p_2}, \\ \varphi (x_2-x_1) H_2 \varphi (x_2-x_1)^{-1}&= e^{2\pi b(p_1+p_2)}. \end{aligned}$$

For the remainder of this section, we let ${\varvec{n}}, \varvec{{{\tilde{n}}}} \in \mathbb {Z}^2$ be a pair of generalized partitions, each with 2 parts. That is

6.2 $$\begin{aligned} \begin{aligned} {\varvec{n}}&= (n_1,n_2), \qquad n_1 \leqslant n_2,\\ \varvec{{{\tilde{n}}}}&= ({{\tilde{n}}}_1, {{\tilde{n}}}_2), \qquad {{\tilde{n}}}_1 \leqslant {{\tilde{n}}}_2. \end{aligned} \end{aligned}$$

We also fix a notation

6.3 $$\begin{aligned} c^\pm _{r,s} = \pm \frac{1}{2}c_b -irb-isb^{-1}. \end{aligned}$$

#### Definition 6.1

The ${\mathfrak {gl}}_2$ *q*-Whittaker polynomial $W_{{\varvec{n}}}({\varvec{z}};q)$ is defined by

6.4 $$\begin{aligned} W_{{\varvec{n}}}({\varvec{z}}; q) = \sum _{k=0}^{n_2-n_1}\left( {\begin{array}{c}n_2-n_1\\ k\end{array}}\right) _q z_1^{n_1+k}z_2^{n_2-k}, \end{aligned}$$

with the *q*-binomial coefficient given by (A.16).

#### Theorem 6.2

The value of the function ${{\widetilde{\Psi }}}_{\varvec{\lambda }}({\varvec{x}})$ at the point ${\varvec{x}} = (c^+_{n_1,{{\tilde{n}}}_1},c^-_{n_2,{{\tilde{n}}}_2})$ is given by

6.5 $$\begin{aligned} {{\widetilde{\Psi }}}_{\varvec{\lambda }}(c^+_{n_1,{{\tilde{n}}}_1},c^-_{n_2,{{\tilde{n}}}_2}) = W_{{\varvec{n}}}(e^{2\pi b \lambda _1},e^{2\pi b \lambda _2};q^{-2}) W_{\varvec{\tilde{n}}}(e^{2\pi b^{-1} \lambda _1},e^{2\pi b^{-1} \lambda _2};{{\tilde{q}}}^{-2}), \end{aligned}$$

where $${{\tilde{q}}} = e^{\pi i b^{-2}}$$.

#### Proof

Let us first consider the value of ${{\widetilde{\Psi }}}_{\varvec{\lambda }}({\varvec{x}})$ at the point

$$\begin{aligned} {\varvec{x}} = (c^+_{n_1,{{\tilde{n}}}_1},c^-_{n_2,{{\tilde{n}}}_2}-\epsilon ) \end{aligned}$$

for some $\epsilon \in \mathbb {R}$. Recalling the poles and zeros of the non-compact dilogarithm (A.3), we see that the downward and upward running sequences of poles of the integrand consist, respectively, of the points

$$\begin{aligned} t^-_{l,{{\tilde{l}}}} = -ilb-i{{\tilde{l}}} b^{-1} \qquad {\text {and}}\qquad t^+_{m,{{\tilde{m}}}} = \epsilon +ib(m-n)+ib^{-1}({{\tilde{m}}}-{{\tilde{n}}}) \end{aligned}$$

where

$$\begin{aligned} n = n_2-n_1, \qquad {{\tilde{n}}} = {{\tilde{n}}}_2 - {{\tilde{n}}}_1, \qquad {\text {and}}\qquad l, {{\tilde{l}}}, m, {{\tilde{m}}} \in \mathbb {Z}_{\geqslant 0}. \end{aligned}$$

Recall now that the integration contour *C* in (2.11) is defined so as to cut the plane into two connected components, one containing the sequence $\{t^-_{l,{{\tilde{l}}}}\}$ and the other the sequence $\{t^+_{m,{{\tilde{m}}}}\}$.

As $\epsilon \rightarrow 0$, two phenomena simultaneously occur: The prefactor $\varphi (x_2-x_1)$ tends to zero, while the $(n+1)(\tilde{n}+1)$ poles of the integrand with

$$\begin{aligned} l+m=n \qquad {\text {and}}\qquad {{\tilde{l}}}+{{\tilde{m}}}={{\tilde{n}}} \end{aligned}$$

collide. Let us write *I* for the integral obtained by pushing the contour of integration *C* across the colliding poles $t^+_{m,\tilde{m}}$, so that setting ${{\widetilde{\Psi }}}_{\varvec{\lambda }}= \int _C{{\tilde{\psi }}}_{\varvec{\lambda }}$, we have

$$\begin{aligned} {{\widetilde{\Psi }}}_{\varvec{\lambda }} = I + 2\pi i\sum _{m=0}^n\sum _{\tilde{m}=0}^{{{\tilde{n}}}}\textrm{Res}_{t=t^+_{m,{{\tilde{m}}}}}{{\tilde{\psi }}}_{\varvec{\lambda }}. \end{aligned}$$

We also have $\lim _{\epsilon \rightarrow 0}I=0$ owing to the vanishing of $\varphi (x_2-x_1)$. Finally, using (A.4) and (A.7) to calculate the sum of $(n+1)({{\tilde{n}}}+1)$ residues, we arrive at the equality (6.5). $\square$

### Macdonald polynomials from Hallnäs–Ruijsenaars functions

The derivation of Macdonald polynomials goes along the similar lines as that of Whittaker polynomials.

#### Definition 6.3

Given a partition ${\varvec{n}}$ as in (6.2), the symmetric ${\mathfrak {gl}}_2$ Macdonald polynomial is defined by

$$\begin{aligned} P_{{\varvec{n}}}({\varvec{x}}; t,q) = \sum _{r=0}^{n_2-n_1} \frac{\left( q^{n_2-n_1};q^{-1}\right) _r \left( t;q\right) _r}{\left( q^{n_2-n_1-1}t;q^{-1}\right) _r \left( q;q\right) _r} x_1^{n_1+r} x_2^{n_2-r}, \end{aligned}$$

where $(X;q)_n$ denotes the *q*-Pochhammer symbol, see (A.15).

Consider the renormalized Hallnäs–Ruijsenaars function

6.6 $$\begin{aligned} {{\widetilde{\Phi }}}^\tau _{\varvec{\mu }}({\varvec{\lambda }}) = \zeta e^{-4\pi i \mu \tau _-} \frac{\varphi (-2\mu -c_b)}{\varphi (-2\mu -2\tau )} \textrm{P}^\tau _{\varvec{\mu }}({\varvec{\lambda }}). \end{aligned}$$

#### Theorem 6.4

Given a pair of partitions $\varvec{n}$, $\varvec{{{\tilde{n}}}}$ as in (6.2), the value of the function ${{\widetilde{\Phi }}}^\tau _{\varvec{\mu }}({\varvec{\lambda }})$ at the point ${\varvec{\mu }} = (-\tau -c^+_{n_1,{{\tilde{n}}}_1},\tau -c^-_{n_2,{{\tilde{n}}}_2})$ is given by the product

6.7 $$\begin{aligned} P_{{\varvec{n}}}(e^{2\pi b\lambda _1}, e^{2\pi b\lambda _2}; e^{2\pi b(2\tau +c_b)}, e^{2\pi i b^2}) P_{\varvec{{{\tilde{n}}}}}(e^{2\pi b^{-1}\lambda _1}, e^{2\pi b^{-1}\lambda _2}; e^{2\pi b^{-1}(2\tau +c_b)}, e^{2\pi i b^{-2}}), \end{aligned}$$

where $c^{\pm }_{r,s}$ is defined by (6.3).

#### Proof

We show the conclusion of the theorem holds for $\tau _-\ne 0$, with the $\tau _-=0$ case then following from the analyticity of both sides in $\tau$. As in the proof of Theorem 6.2, we consider the value of ${{\widetilde{\Phi }}}^\tau _{\varvec{\mu }}({\varvec{\lambda }})$ at

$$\begin{aligned} {\varvec{\mu }} = (-\tau -c^+_{n_1,{{\tilde{n}}}_1}-\varepsilon ,\tau -c^-_{n_2,\tilde{n}_2}+\varepsilon ). \end{aligned}$$

As $\varepsilon \rightarrow 0$, we see that the zeros

$$\begin{aligned} x^+_{m,{{\tilde{m}}}} = ib\left( \frac{n_2-n_1}{2}-m\right) + ib\left( \frac{\tilde{n}_2 - {{\tilde{n}}}_1}{2}-{{\tilde{m}}}\right) + \varepsilon , \qquad m, {{\tilde{m}}} \in \mathbb {Z}_{\geqslant 0} \end{aligned}$$

of the dilogarithm $\varphi (x-\mu -\tau _-)$ collide with the poles

$$\begin{aligned} x^-_{l,{{\tilde{l}}}} = ib\left( l-\frac{n_2-n_1}{2}\right) + ib\left( {{\tilde{l}}} - \frac{{{\tilde{n}}}_2 - {{\tilde{n}}}_1}{2}\right) - \varepsilon , \qquad l, {{\tilde{l}}} \in \mathbb {Z}_{\geqslant 0} \end{aligned}$$

of the dilogarithm $\varphi (x+\mu +\tau _-)$. Following the reasoning in the proof of Theorem 6.2, we obtain

$$\begin{aligned} {{\widetilde{\Phi }}}^\tau _{\varvec{\mu }} = -2\pi i\sum _{m=0}^{n_2-n_1} \sum _{{{\tilde{m}}}=0}^{{{\tilde{n}}}_2 - \tilde{n}_1}\textrm{Res}_{x=x^+_{m,{{\tilde{m}}}}}{{\tilde{\phi }}}_{\varvec{\mu }}^\tau , \end{aligned}$$

where ${{\widetilde{\Phi }}}^\tau _{\varvec{\mu }} = \int _C \tilde{\phi }_{\varvec{\mu }}^\tau$. Finally, the evaluation of residues yields the desired result. $\square$

### Harish–Chandra series from Whittaker functions

By the convention (2.3), the contour *C* in the integral (6.1) passes above the zeros of $\varphi (t-c_b)$, below those of $\varphi (x_2-x_1-t)$, and can be chosen to escape to infinity along the real line. The zeros of the factor $\varphi (t-c_b)$ are at $t=t_{r,s}=-i(br+b^{-1}s)$, $r,s\in \mathbb {Z}_{\geqslant 0}$. Using equations (A.4) and (A.6), we find that

$$\begin{aligned}  &   \textrm{Res}_{t=t_{r,s}} \left( e^{2\pi it(\lambda _1-\lambda _2)}\frac{\varphi (x_2-x_1)}{\varphi (t-c_b)\varphi (x_2-x_1-t)}\right) \\  &   \qquad \quad = -\zeta \Lambda _{1,2}^r {{\widetilde{\Lambda }}}_{1,2}^s \frac{(-qX_{2,1};q^2)_r}{(q^{-2r};q^{2})_r} \frac{(-q\widetilde{X}_{2,1};{{\tilde{q}}}^2)_s}{({{\tilde{q}}}^{-2r};{{\tilde{q}}}^{2})_s}, \end{aligned}$$

where $X_j=e^{2\pi bx_j}$, ${{\widetilde{X}}}_j=e^{2\pi b^{-1} x_j}$, and we write $A_{j,k}$ for the ratio $A_j/A_k$. Making a change of variables

$$\begin{aligned} x_1'=x_1-\frac{c_b}{2}, \qquad x_2'=x_2+\frac{c_b}{2} \end{aligned}$$

and setting $X_j'=e^{2\pi b x_j'}$, we arrive at the (asymptotically free) Harish–Chandra series for the ${\mathfrak {gl}}_2$ Whittaker *q*-difference equation, see, e.g., [7, 8],^2^

6.8 $$\begin{aligned} \sum _{r \in \mathbb {Z}_{\geqslant 0}}\textrm{Res}_{t=t_{r,0}}(\tilde{\psi }) = \Lambda _1^{ib^{-1}x'_1} \Lambda _2^{ib^{-1}x'_2} \sum _{r\geqslant 0} \Lambda _{1,2}^r \frac{(X'_{2,1};q^2)_r}{(q^{-2r};q^2)_r}. \end{aligned}$$

By d’Alembert’s ratio test, the above series has infinite radius of convergence if $|q|<1$ and zero radius of convergence if $|q|>1$. One can also check that a similar calculation with the zeros of the factor $\varphi (x_2-x_1-t)$ yields an expression (6.8) with $\Lambda _1$ and $\Lambda _2$ interchanged. Finally, we note that conditions $x_1=c^+_{n_1,\tilde{n}_1}$, $x_2=c^-_{n_2,{{\tilde{n}}}_2}$ imply $X_{2,1}'=q^{2(n_1-n_2)}$, and the Harish–Chandra series (6.8) truncates to the first factor in (6.5).

### Harish–Chandra series from Hallnäs–Ruijsenaars functions

Similarly to the Whittaker case, we shall calculate the sum of the residues of the integrand in the expression (2.5) at points

$$\begin{aligned} x_{r,0}^{\pm } = \pm \mu -\tau + c_{r,0}^-, \end{aligned}$$

where $\mu = (\mu _1-\mu _2)/2$ and $c_{r,s}^-$ is defined by (6.3). Let us denote

$$\begin{aligned} \mu '_1 = \mu _1-\tau _+, \qquad \mu '_2 = \mu _2+\tau _+, \qquad {\text {and}}\qquad \mu ''_j = -ib^{-1}\mu '_j \end{aligned}$$

for $j=1,2$. Then, the contribution $\textrm{P}_+$ from the poles at $x=x_{r,0}^+$ reads:

$$\begin{aligned} \textrm{P}_+ = \zeta \zeta _{inv} e^{4\pi i \tau ^2} e^{2\pi i \tau _-(\mu '_2-\mu '_1)} \frac{\varphi (\mu _1-\mu _2-2\tau )}{\varphi (\mu _1-\mu _2-c_b)} {{\varvec{\Lambda }}}^{{\varvec{\mu }}''} P_{q,t}(\Lambda _{2,1}\vert \textrm{M}_{1,2}), \end{aligned}$$

where

$$\begin{aligned} {\varvec{\Lambda }}^{{\varvec{\mu }}''} = \Lambda _1^{\mu ''_1} \Lambda _2^{\mu ''_2} \qquad {\text {and}}\qquad P_{q,t}(\Lambda \vert \textrm{M}) = \sum _{r \geqslant 0} \Lambda ^r \frac{(t^{-2}\textrm{M};q^{-2})_r (t^2;q^2)_r}{(q^{-2}\textrm{M};q^{-2})_r (q^2;q^2)_r}. \end{aligned}$$

By d’Alembert’s ratio test, the radius of convergence of the series $P_{q,t}$ is $\left| t^2/q^2\right|$ if $|q|<1$ and $\left| q^2/t^2\right|$ if $|q|>1$. We note that the product ${{\varvec{\Lambda }}}^{\varvec{\mu ''}} P_{q,t}(\Lambda _{2,1}\vert \textrm{M}_{1,2})$ is the Harish–Chandra series solution to the Macdonald eigenvalue equation, see [7, 24, 30]. It is immediate to see that the contribution $\textrm{P}_-$ from the poles at $x=x_{r,0}^-$ can be obtained from $\textrm{P}_+$ by swapping $\mu _1$ and $\mu _2$.

#### Remark 6.5

One can now see that the series, obtained from (2.5) by computing residues at all poles of the integrand of the form $\pm \mu -\tau + c_{r,s}^-$, is proportional (up to a function of ${\varvec{\mu }}$) to the series

$$\begin{aligned} e^{-2 \pi i {\varvec{\lambda }} \cdot {\varvec{\mu }}} e^{2 \pi i \tau _+(\lambda _1-\lambda _2)} P_{q,t}\left( e^{2 \pi b (\lambda _2-\lambda _1)}\Big \vert e^{2 \pi b (\mu _1-\mu _2)}\right) P_{{{\tilde{q}}},{{\tilde{t}}}}\left( e^{2 \pi b^{-1} (\lambda _2-\lambda _1)}\Big \vert e^{2 \pi b^{-1} (\mu _1-\mu _2)}\right) , \end{aligned}$$

which has a nonzero radius of convergence when $b \notin \mathbb {R}$. More precisely, the radius is equal to

$$\begin{aligned} R = {\left\{ \begin{array}{ll} \min \left( \left| t^2/q^2\right| , \left| {{\tilde{q}}}^2/{{\tilde{t}}}^2\right| \right) , & {\text {if}} \; |q|<1, \\ \min \left( \left| q^2/t^2\right| , \left| {{\tilde{t}}}^2/{{\tilde{q}}}^2\right| \right) , & {\text {if}} \; |q|>1, \end{array}\right. } \end{aligned}$$

where we set

$$\begin{aligned} {{\tilde{t}}} = e^{\pi b^{-1} (2\tau +c_b)}. \end{aligned}$$

Finally, evaluating the expression

6.9 $$\begin{aligned} \zeta e^{-4\pi i \mu \tau _-} \frac{\varphi (-2\mu -c_b)}{\varphi (-2\mu -2\tau )} \left( \textrm{P}_+ + \textrm{P}_-\right) \end{aligned}$$

at point ${\varvec{\mu }} = (-\tau -c^+_{n_1,\tilde{n}_1},\tau -c^-_{n_2,{{\tilde{n}}}_2})$, we see that the first summand vanishes, since the dilogarithm $\varphi (-2\mu -2\tau )$ has a pole, while the second summand truncates to the first factor in (6.7).

## Conclusion

Let us start by recalling our strategy for deriving the joint eigenfunctions for the Macdonald–Ruijsenaars operators $M_j^\tau$, with $j=1,2$. We first observe that these operators are obtained from the action of particular elements of the spherical DAHA ${\mathbb{S}\mathbb{H}}_{q,t}(GL_2)$ in a Hilbert space representation of the latter. Using cluster theory, we show that this representation carries an action of $SL(2,\mathbb {Z})$ by unitary automorphisms, which intertwine the $SL(2,\mathbb {Z})$ action on ${\mathbb{S}\mathbb{H}}_{q,t}(GL_2)$ and can be computed explicitly. From this, we deduce that the action of the element $S\in SL(2;{\mathbb {Z}})$ intertwines the action of the Macdonald–Ruijsenaars operators with that of the multiplication operators $e_j({\varvec{\lambda }})$ and so delivers the sought-for unitary joint eigenfunction transform for the $M_j^\tau$.

Our explicit computation of the action of the modular group element *S* hinges on the identification of the spherical DAHA ${\mathbb{S}\mathbb{H}}_{q,t}(GL_2)$ with the quantized coordinate ring of the moduli space of decorated $GL_2({\mathbb {C}})$-local systems on the punctured torus. Indeed, by the work of Fock and Goncharov, the corresponding classical moduli space carries a cluster structure, on which the mapping class group $SL(2,{\mathbb {Z}})$ acts by cluster transformations.

Generalizing our approach to the *N*-particle case requires identifying the spherical DAHA ${\mathbb{S}\mathbb{H}}_{q,t}(GL_N)$ with the quantized coordinate ring of an appropriate cluster variety. The variety in question parametrizes $GL_N(\mathbb {C})$-local systems on the punctured torus, whose monodromy *g* around the puncture satisfies $\operatorname {rank}(g-t\operatorname {Id}) \leqslant 1$, equipped with a flat section of the associated $\mathbb {P}^{N-1}$-bundle near the puncture. Although for $N>2$ this constrained moduli space does not fall within the Fock–Goncharov construction, it nonetheless admits a cluster parametrization in which the mapping class group $SL(2,\mathbb {Z})$ again acts by cluster transformations. The combinatorics of this action is much more complicated than in the $N=2$ case, however, making it a challenge to write explicit formulas for integral kernels such as the ones used in this paper to describe the action of *S*.

Despite this difficulty, there are potential advantages offered by the richer combinatorics in higher rank. Indeed, for $N=2$ the mutable parts of all quivers in the cluster atlas on the moduli space coincide with the Markov quiver, while for $N>2$ the corresponding quivers are of infinite mutation type. Each mutation class of the quiver affords a different factorization of the action of *S* into cluster transformations, and hence a different presentation for the joint eigenfunctions. We hope that this plethora of equivalent representations can prove useful in the study of the eigenfunctions, and hope to return to the issue in a subsequent publication.

## Data Availability

Data sharing is not applicable to this article as no datasets were generated or analyzed during the current study.

## Quantum dilogarithms

In this Appendix, we recall the definition and some properties of the quantum dilogarithm, mostly following [22] and [13].

### Non-compact quantum dilogarithm

#### Definition A.1

The *non-compact quantum dilogarithm function* $\varphi _b(z)$ is defined in the strip $\left| \operatorname {Im}(z)\right| < \left| \operatorname {Im}(c_b)\right|$ by the following formula:

$$\begin{aligned} \varphi _b(z) = \exp \left( \frac{1}{4} \int _C\frac{e^{-2izt}}{\operatorname {sh}(tb)\operatorname {sh}(tb^{-1})}\frac{dt}{t}\right) , \end{aligned}$$

where the contour *C* follows the real line from $-\infty$ to $+\infty$, surpassing the origin in a small semi-circle from above.

The non-compact quantum dilogarithm can be analytically continued to the complex plane as a meromorphic function with an essential singularity at infinity. The resulting function, which we denote by the same symbol $\varphi _b(z)$, enjoys the symmetry

$$\begin{aligned} \varphi _b(z) = \varphi _{-b}(z) = \varphi _{b^{-1}}(z). \end{aligned}$$

In what follows, we shall write $\varphi (z)$ for $\varphi _b(z)$, omitting the symbol *b* from the notation. If $b+b^{-1}\in {\mathbb {R}}$, then the function $\varphi$ satisfies the unitarity relation

A.1 $$\begin{aligned} \overline{\varphi (z)} \varphi ({\overline{z}}) = 1. \end{aligned}$$

We assume $b+b^{-1} \in \mathbb {R}_{>0}$ and recall the pure imaginary constant

$$\begin{aligned} c_b = \frac{i(b + b^{-1})}{2} \end{aligned}$$

along with phase constants

A.2 $$\begin{aligned} \zeta = e^{\pi i(1-4c_b^2)/12} \qquad {\text {and}}\qquad \zeta _{\textrm{inv}} = \zeta ^{-2} e^{-\pi i c_b^2}. \end{aligned}$$

Then, the poles and zeros of $\varphi (z)$ are given by

A.3 $$\begin{aligned} \varphi (z)^{\pm 1} = 0 \quad \Leftrightarrow \quad z = \mp \left( c_b + ibm + ib^{-1}n\right) \quad {\text {for}}\quad m,n \in \mathbb {Z}_{\geqslant 0}. \end{aligned}$$

The asymptotic behavior around its zeros, poles, and infinity is described by

A.4 $$\begin{aligned} \varphi (z\pm c_b) \sim \pm \zeta ^{-1} (2 \pi i z)^{\mp 1} \qquad {\text {as}}\qquad z \rightarrow 0, \end{aligned}$$

and

A.5 $$\begin{aligned} \varphi (z) \sim {\left\{ \begin{array}{ll} \zeta _{\textrm{inv}} e^{\pi i z^2}, &  \operatorname {Re}(z) \rightarrow +\infty , \\ 1, &  \operatorname {Re}(z) \rightarrow -\infty , \end{array}\right. } \end{aligned}$$

respectively. Finally, $\varphi$ satisfies the reflection/inversion relation,

A.6 $$\begin{aligned} \varphi (z) \varphi (-z) = \zeta _{\textrm{inv}} e^{\pi i z^2}, \end{aligned}$$

as well as the functional equations

A.7 $$\begin{aligned} \varphi \left( z - ib^{\pm 1}/2\right) = \left( 1 + e^{2\pi b^{\pm 1}z}\right) \varphi \left( z + ib^{\pm 1}/2\right) , \end{aligned}$$

and the pentagon identity

A.8 $$\begin{aligned} \varphi (p) \varphi (x) = \varphi (x) \varphi (p+x) \varphi (p), \end{aligned}$$

which holds for any pair of self-adjoint operators *p* and *x* satisfying $[p,x] = \frac{1}{2\pi i}$.

The non-compact quantum dilogarithm is closely related to the Ruijsenaars hyperbolic Gamma function $G(\omega _1, \omega _2; z)$ used in works [19–21], and the Barnes double sine function $S_2(z) = S_2(z \,|\,\omega _1,\omega _2)$ used in [1–4]. The latter is defined by the formula

A.9 $$\begin{aligned} S_2(z) = \exp \left( \int _0^\infty \left( \frac{\operatorname {sh}((2z-\omega _1-\omega _2)t)}{\operatorname {sh}(\omega _1t)\operatorname {sh}(\omega _2t)} - \frac{2z-\omega _1-\omega _2}{\omega _1\omega _2t}\right) \frac{dt}{2t}\right) \end{aligned}$$

and satisfies the relation

$$\begin{aligned} S_2\!\left( z \,\big |\, b,b^{-1}\right) = \varphi (iz-c_b) e^{-\frac{\pi i}{2}(iz-c_b)^2}. \end{aligned}$$

The former can be expressed as

$$\begin{aligned} G(\omega _1, \omega _2; z) = S_2\left( iz+\frac{\omega _1+\omega _2}{2} \,\bigg |\, \omega _1, \omega _2\right) . \end{aligned}$$

### Integral identities

The quantum dilogarithm function $\varphi (z)$ satisfies many integral identities, all of which make use of the convention (2.3). Namely, in any contour integral of the form

$$\begin{aligned} \int _{C} \prod _{j,k}\frac{\varphi (t-a_j)}{\varphi (t-b_k)}f(t)dt, \end{aligned}$$

where *f*(*t*) is some entire function, we assume that the contour *C* is passing below the poles of $\varphi (t-a_j)$ for all *j*, above the poles of $\varphi (t-b_k)^{-1}$ for all *k*, and escaping to infinity in such a way that the integrand is rapidly decaying.

The Fourier transform of the quantum dilogarithm can be calculated explicitly:

A.10 $$\begin{aligned} \zeta \varphi (w)&= \int \frac{e^{2\pi i x(w-c_b)}}{\varphi (x-c_b)} dx, \end{aligned}$$

A.11 $$\begin{aligned} \frac{1}{\zeta \varphi (w)}&= \int \frac{\varphi (x+c_b)}{e^{2\pi i x(w+c_b)}} dx. \end{aligned}$$

Note that in accordance with Notation 2.3, the integration contours in (A.10) and (A.11) can be taken to be $\mathbb {R}+i\varepsilon$ and $\mathbb {R}-i\varepsilon$, respectively, with a small positive number $\varepsilon$.

Using the above Fourier transform to compute the integral kernels for the operators $\varphi (p)$ and $\varphi (p+x)$, one sees that the pentagon identity (A.8) is equivalent to either of the following integral analogs of Ramanujan’$\hbox {s}_{~1\psi _{1}}$ summation formula:

A.12 $$\begin{aligned} \int \frac{\varphi (x+u)}{\varphi (x+v)} e^{2\pi i xw} dx&= \zeta e^{-2\pi i w(v+c_b)} \frac{\varphi (u-v-c_b) \varphi (w+c_b)}{\varphi (u-v+w-c_b)}, \end{aligned}$$

A.13 $$\begin{aligned}&= \zeta ^{-1} e^{-2\pi i w(u-c_b)} \frac{\varphi (v-u-w+c_b)}{\varphi (v-u+c_b) \varphi (-w-c_b)}. \end{aligned}$$

Finally, we will use the following limit of the integral analogue of the Saalschütz summation formula:

A.14 $$\begin{aligned}&\int e^{-4\pi i c_b x} \frac{\varphi (x+u_1) \varphi (x+u_2)}{\varphi (x+v-c_b)\varphi (x-c_b)} dx\nonumber \\&\quad = \zeta ^3 e^{\pi i v(2c_b-v)}\frac{\varphi (u_1)\varphi (u_2)\varphi (u_1-v)\varphi (u_2-v)}{\varphi (u_1+u_2-v-c_b)}. \end{aligned}$$

### Compact quantum dilogarithm

Recall the standard notation for the *q*-Pochhammer symbol:

A.15 $$\begin{aligned} (X;q)_n = \prod _{k=0}^{n-1}(1-q^kX). \end{aligned}$$

so that the *q*-binomial coefficient can be expressed as

A.16 $$\begin{aligned} \left( {\begin{array}{c}n\\ k\end{array}}\right) _q = \frac{(q;q)_n}{(q;q)_k(q;q)_{n-k}}. \end{aligned}$$

The *compact quantum dilogarithm function* $\Psi _q(X)$ is defined by the series expansion of

A.17 $$\begin{aligned} \Psi _q(X) = \prod _{n\geqslant 0}\left( 1+Xq^{2n+1}\right) ^{-1} \in {\mathbb {Q}}(q)[[X]], \end{aligned}$$

or equivalently

$$\begin{aligned} \Psi _q(X) = (-qX;q^2)_{\infty }^{-1}. \end{aligned}$$

For $|q|<1$, the product (A.17) is convergent, and for $\operatorname {Im}(b^2)>0$ the compact and non-compact versions of the quantum dilogarithm function are related by

$$\begin{aligned} \varphi _b(z) = \frac{\Psi _{{{\tilde{q}}}^{-1}}\big (e^{2\pi b^{-1}z}\big )}{\Psi _{q}\big (e^{2\pi bz}\big )}, \qquad {\text {where}}\qquad q = e^{\pi i b^2}, \quad {{\tilde{q}}} = e^{\pi i b^{-2}}. \end{aligned}$$

The analog of the functional equation (A.7) is given by

A.18 $$\begin{aligned} \Psi _q(qX) = (1+X)\Psi _q(q^{-1}X). \end{aligned}$$

We will use the following discrete (or “compact”) analogs of the integral identities from the previous section. A discrete analog of the Fourier transform formula (A.10) is given by the Taylor series expansion of the Pochhammer symbol:

$$\begin{aligned} (z;q)^{-1}_{\infty } = \sum _{n\geqslant 0}\frac{z^n}{(q;q)_n}, \end{aligned}$$

or more suggestively

$$\begin{aligned} \frac{(q;q)_\infty }{(z;q)_{\infty }} = \sum _{n\geqslant 0}{(q^{n+1};q)_\infty }z^n, \end{aligned}$$

with the constant $(q;q)_\infty$ playing the role of phase $\zeta$ in (A.10). A discrete analog of the pentagon integral (A.12) is given by the *q*-binomial theorem

$$\begin{aligned} \sum _{n\geqslant 0}\frac{(a;q)_n}{(q;q)_n}z^n = \frac{(az;q)_{\infty }}{(z;q)_{\infty }}, \quad |z|<1, \quad |q|<1, \end{aligned}$$

or equivalently

$$\begin{aligned} \sum _{n\geqslant 0} \frac{(q^{n+1};q)_\infty }{(aq^{n};q)_\infty } = (q;q)_\infty \cdot \frac{(az;q)_{\infty }}{(a;q)_\infty (z;q)_{\infty }}. \end{aligned}$$

As for the integral (A.14), in terms of the *q*-hypergeometric series

we have Heine’s *q*-analogue of Gauss’ summation formula:

A.19

Heine’s formula can also be written in the following form more reminiscent of (A.14):

$$\begin{aligned} \sum _{n\geqslant 0} \frac{(c q^n,q)_\infty (q^{n+1},q)_\infty }{(aq^n,q)_\infty (bq^n,q)_\infty } z^n = (q,q)_\infty \frac{(c/a,q)_\infty (c/b,q)_\infty }{(a,q)_\infty (b,q)_\infty (c/(a b),q)_\infty }, \end{aligned}$$

In the special case $b = q^{-n}$ with $n\in {\mathbb {Z}}_{\geqslant 0}$, the right-hand side of (A.19) terminates and reduces to the Chu–Vandermonde formula

A.20

## Footnotes

1 Note that our notations differ from those in [2]. Namely, $M_1$ and $M_2$ in *loc. cit.* are, respectively, $M_1 M_2^{-1}$ and $M_2^{-1}$ here.

2 Note that in *loc.cit.* variables $\Lambda$ and *X* are interchanged.
